# Fluorinated Alkyl Chains Terminated Polar Glycol Ether Side Chain for N‐Type Organic Thermoelectrics with Enhanced Performance and Air Stability

**DOI:** 10.1002/advs.202500571

**Published:** 2025-04-07

**Authors:** Gang Ye, Yazhuo Kuang, Mingyu Ma, Xiantao Peng, Jian Liu

**Affiliations:** ^1^ Key Laboratory for the Green Preparation and Application of Functional Materials Hubei Key Laboratory of Polymer Materials School of Materials Science and Engineering Hubei University Youyi Road 368 Wuhan 430062 P. R. China; ^2^ State Key Laboratory of Polymer Science and Technology Changchun Institute of Applied Chemistry Chinese Academy of Sciences Changchun 130022 P. R. China; ^3^ School of Applied Chemistry and Engineering University of Science and Technology of China Hefei 230026 P. R. China

**Keywords:** air stability, fluorinated alkyl chains, n‐type conjugated polymer, organic thermoelectrics, polar side chain

## Abstract

N‐type organic thermoelectric materials with excellent device performance and air stability are critically important for practical applications in organic thermoelectric devices. However, the design and synthesis of air‐stable n‐type conductive materials with high electrical conductivities remain a significant challenge. In this paper, a new strategy is reported to enhance the thermoelectric performance and air stability of naphthalenediimide (NDI)‐dialkoxybithiazole‐based conjugated polymers by introducing fluorinated alkyl chains terminated polar glycol ether side chains into the NDI unit. After molecular doping with 4‐(2,3‐dihydro‐1,3‐dimethyl‐1H‐benzoimidazol‐2‐yl) phenyl)‐N, N‐dimethylbenzenamine (N‐DMBI), the doped films of the fluorinated alkyl side‐chain modified conjugated polymer exhibit higher electrical conductivity, thus resulting in a greater power factor; and better air stability compared to those of the unmodified polymer with the same conjugated backbone. The work provides insights into material design for the future development of air‐stable n‐type organic thermoelectrics.

## Graphical abstract

Conjugated polymers containing a naphthalenediimide‐dialkoxybithiazole backbone, featuring with different fluorinated alkyl chains terminated the oligo (ethylene glycol) side chains, are designed and synthesized for n‐type organic thermoelectrics. This new side chain strategy not only ensures the solubility of dopant molecules in the host matrix but also enhances the water‐repellent properties, thereby keeping off water and enabling applications in organic thermoelectric materials with boosted performance and improved air stability.

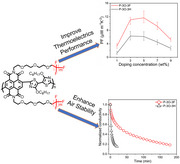

## 1 Introduction

Thermoelectric materials, which enable direct energy conversion from natural or waste heat resources to electricity, have been widely used in energy generators and other multiple applications.^[^ ^1^, ^2^, ^3^, ^4^, ^5^ ^]^ Organic thermoelectric (OTE) materials have recently received renewed attention because of their attractive features, such as lightweight, high mechanical flexibility, low toxicity, low‐cost energy generation, and solution processibility.^[^ ^6^, ^7^, ^8^, ^9^, ^10^, ^11^, ^12^, ^13^, ^14^, ^15^ ^]^ In particular, the properties of organic thermoelectric materials can be easily and precisely tuned by exploiting their chemical structures. The dimensionless figure of merit (*ZT* = *S* ^2^ *σ*T/*κ*, where *S*, *σ*, T, and *κ* are the Seebeck coefficient, electrical conductivity, absolute temperature, and thermal conductivity, respectively) is quantified to evaluate the thermoelectric performance of thermoelectric materials. Due to OTE materials' intrinsic low thermal conductivities (0.3–1.0 W m^−1^ K^−1^), the power factor (*PF* = *S* ^2^ *σ*) is another metric used to evaluate the thermoelectric performance in conjugated polymers.^[^ ^7^, ^8^, ^16^ ^]^

Both p and n‐type OTE materials with comparable performance are needed to achieve highly efficient thermoelectric power generators.^[^ ^11^, ^17^, ^18^, ^19^ ^]^ However, the thermoelectric performance of n‐type OTE materials lags greatly behind their p‐type counterparts; this unbalanced condition severely restricts their potential applications.^[^ ^20^, ^21^, ^22^, ^23^ ^]^ Therefore, n‐type organic thermoelectric materials with excellent device performance that are air‐stable are critically important for further practical applications.^[^ ^17^ ^]^ In the past decade, we have witnessed the rapid development of n‐dope. d conjugated polymeric thermoelectric materials regarding electrical conductivity and *PF* or *ZT*.^[^ ^17^, ^24^, ^25^, ^26^, ^27^, ^28^, ^29^, ^30^ ^]^ For instance, Bao's group proposed a breakthrough molecular backbone design for n‐type OTE polymers by developing an acceptor–acceptor‐type conjugated polymer rather than the conventional donor–acceptor (D‐A) backbone.^[^ ^31^ ^]^ Following this concept, numerous acceptor–acceptor‐type conjugated polymers exhibiting excellent thermoelectric performance have been developed recently.^[^ ^30^, ^32^, ^33^, ^34^, ^35^, ^36^, ^37^, ^38^, ^39^, ^40^ ^]^ Beyond the backbone engineering, side‐chain engineering is also important in increasing the performance of organic thermoelectric materials due to the enhanced host‐dopant miscibility^[^ ^41^, ^42^, ^43^, ^44^, ^45^, ^46^, ^47^ ^]^ and charge transport properties.^[^ ^17^, ^48^, ^49^ ^]^

The earlier advancements in n‐type OTE materials mainly focused on the advances in the molecular design of polymeric backbone^[^ ^34^, ^35^, ^36^, ^38^, ^50^ ^]^ and side‐chain engineering^[^ ^48^, ^49^ ^]^ to boost their thermoelectric performance. However, less attention has been paid to designing air‐stable n‐type OTE materials.

A common difficulty in employing n‐doped OTE materials is that most of these materials are sensitive to the ambient environment due to the quenching of the charge‐carrying radical anions by H_2_O and O_2_, which significantly hampers their processing, characterization, and application in device fabrication within an ambient condition.^[^ ^4^, ^6^, ^51^, ^52^ ^]^ Access to air‐stable n‐type OTE materials with high electrical conductivity remains challenging.^[^ ^51^, ^52^ ^]^

Recently, few research efforts have focused on enhancing the air stability of n‐type doped conjugated polymers by lowering the lowest unoccupied molecular orbital (LUMO) energy level of the n‐type conjugated polymer^[^ ^53^ ^]^ or by employing a self‐encapsulated thick film strategy.^[^ ^54^, ^55^, ^56^ ^]^

The quenching process of active radical anions in n‐doped conjugated polymers is strongly related to their LUMO energy level.^[^ ^4^, ^51^, ^52^ ^]^ To avoid the annihilation reaction between radical anions and oxygen/water, the LUMO level of n‐type polymers must be below the oxygen and water's redox potentials (around −4.99 eV).^[^ ^52^ ^]^ Therefore, n‐doped conjugated polymers with deeper LUMO levels would exhibit higher air stability than those with less deep LUMO levels in the ambient condition. However, achieving n‐type conjugated polymers with LUMO levels below −4.99 eV is extremely challenging. Until now, only one conjugated polymer has reached this target.^[^ ^53^ ^]^ Fei Huang et al. recently reported an n‐doped conjugated polymer PBFDO with an extremely deep LUMO level (−5.18 eV), exhibiting excellent air stability.^[^ ^53^ ^]^ The electrical conductivity of the unencapsulated polymer film only lost 5% of the initial value after 35 days of exposure to ambient conditions.

Another widely used strategy to avoid quenching decay is self‐encapsulated thick film. For an n‐doped thick film, the outer top layer materials would protect the inner conductive materials by sacrificing themselves to form a blocking layer after reacting with O_2_ and H_2_O, leading to a slower dedoping rate.^[^ ^54^, ^55^, ^56^ ^]^ For example, in 2017, Howard E. Katz et al. reported that the tetra‐n‐butylammonium fluoride (TBAF)‐doped n‐type conjugated polymer ClBDPPV (LUMO = −4.3 eV) exhibits significant air stability, maintaining a conductivity of over 0.1 S cm^−1^ after one week of air exposure in a thick film (around 8 µm).^[^ ^55^ ^]^ Later, Pei et al. reported on an n‐type ladder polymer, LPPV‐1 (LUMO = −4.49 eV), doped with N‐DMBI.^[^ ^56^ ^]^ The electrical conductivity of a micrometer‐thick doped LPPV‐1 film maintained 61% of its initial value and remained at 0.60 S cm^−1^ after 76 days of exposure to air.

Herein, we report a new strategy to enhance the thermoelectric performance and air stability of naphthalenediimide (NDI)‐dialkoxybithiazole‐based conjugated polymers by introducing fluorinated alkyl chains terminated polar glycol ether side chains into the NDI unit. The polar triethylene glycol side chains can ensure good host/dopant miscibility.^[^ ^41^, ^44^, ^45^, ^57^ ^]^ The fluorinated alkyl chains possess excellent water‐repellent properties, potentially serving as a molecular shield to protect against water and rendering them suitable for organic thermoelectric (OTE) materials with improved air stability.^[^ ^58^, ^59^, ^60^, ^61^ ^]^ In addition, the fluorinated alkyl chains can facilitate self‐assembly, enhance charge mobility, and benefit thermoelectric performance.^[^ ^62^, ^63^ ^]^ After molecular doping with 4‐(2,3‐dihydro‐1,3‐dimethyl‐1H‐benzoimidazol‐2‐yl)phenyl)‐N,N‐dimethylbenzenamine (N‐DMBI), the doped films of the fluorinated alkyl side‐chain modified conjugated polymer exhibit higher electrical conductivity, thus a greater power factor, and better air stability than those of the unmodified polymer with the same conjugated backbone. Our work provides insights into material design for the future development of air‐stable n‐type organic thermoelectrics.

## 2 Results and Discussion

### 2.1 Polymer Design, Synthesis, and Characterization

**Figure 1:**
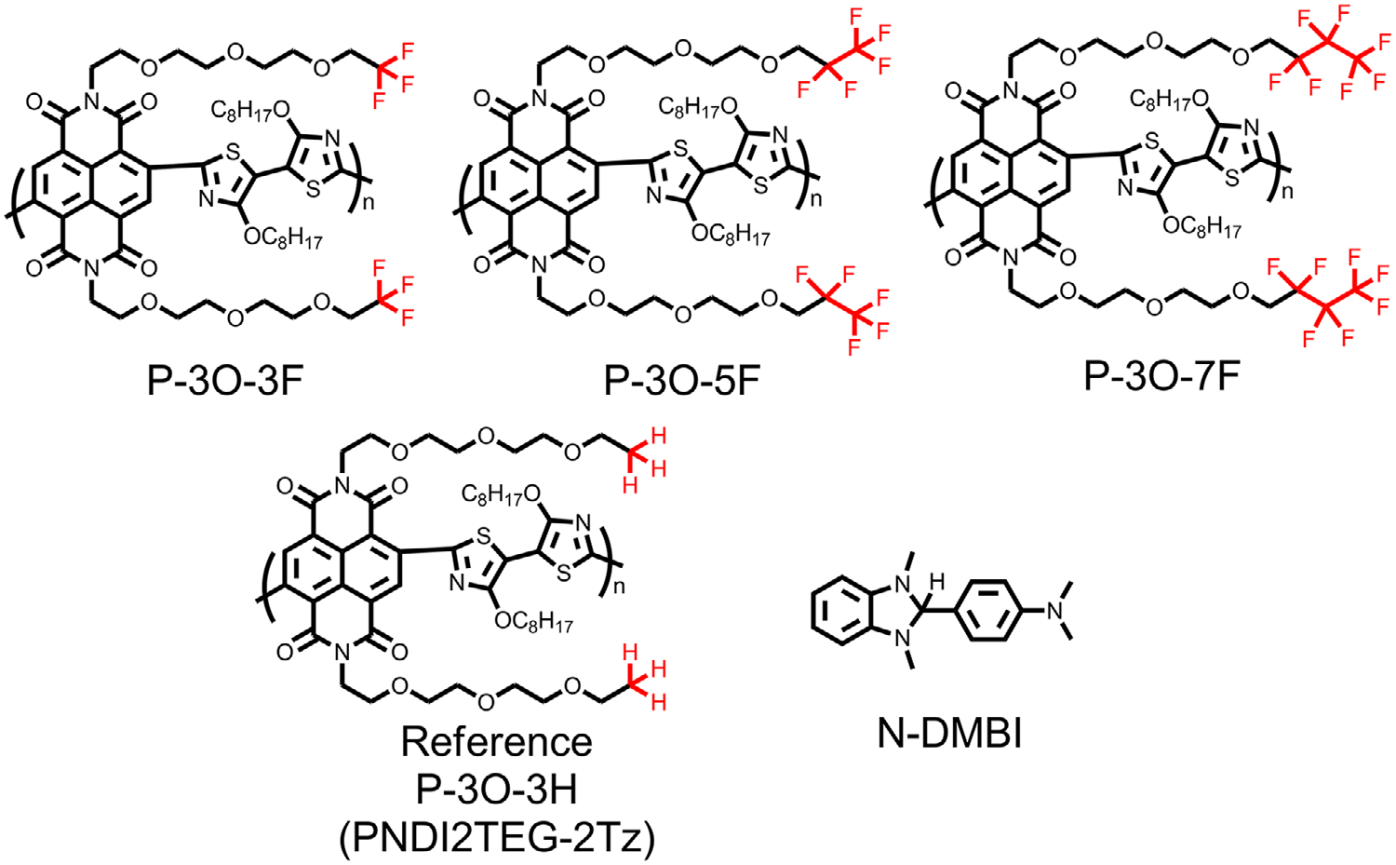
Chemical structures of naphthalenediimide‐dialkoxybithiazole‐based copolymers with different fluorinated alkyl chains terminated OEG side chains and n‐type dopant N‐DMBI.

**Scheme 1:**
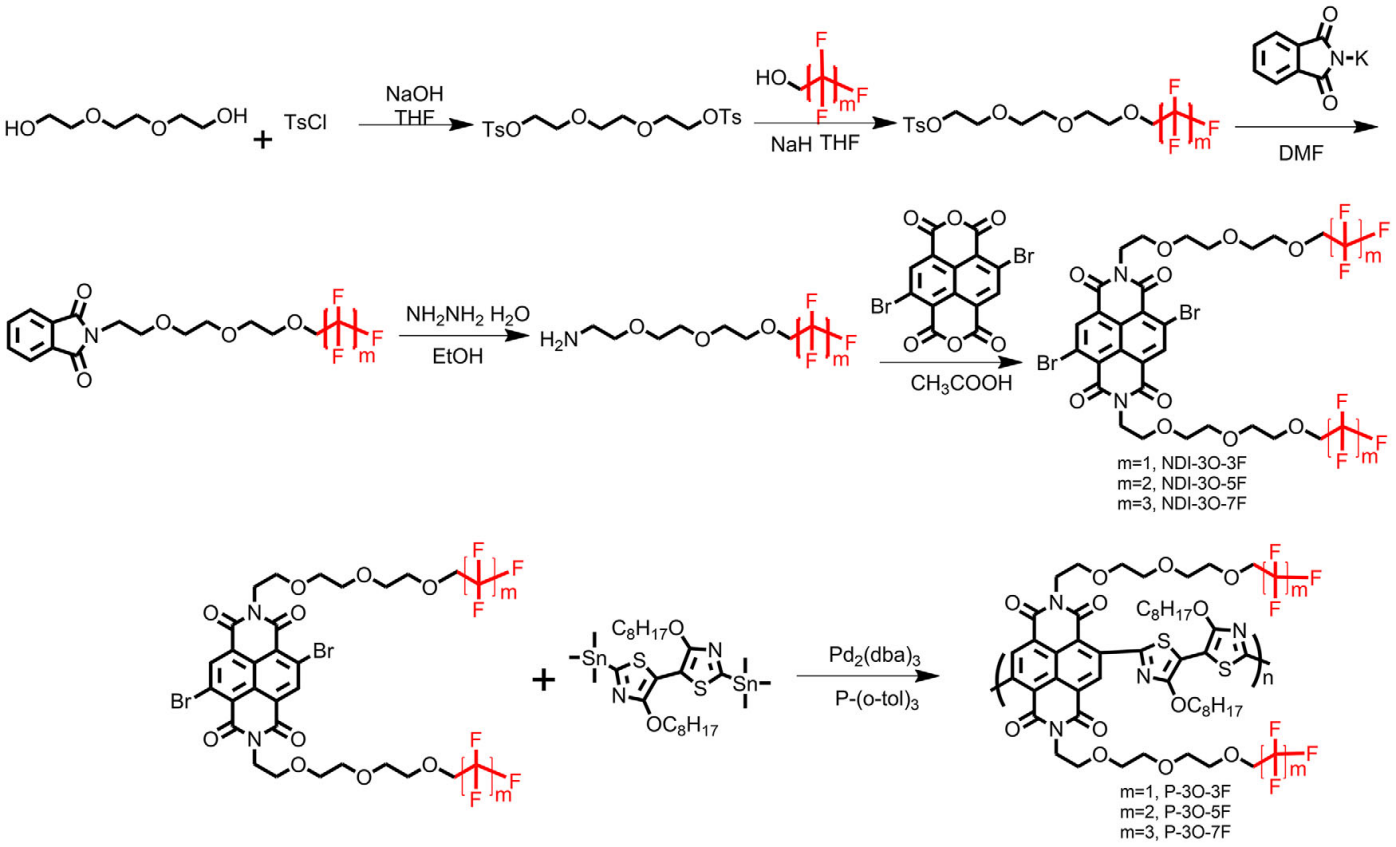
Synthetic route to the naphthalenediimide‐dialkoxybithiazole based conjugated polymer P‐3O‐3F, P‐3O‐5F, and P‐3O‐7F.

**Figure** **1** displays the chemical structures of the NDI‐2Tz‐based copolymers functionalized with different fluorinated alkyl chains terminated OEG (oligo (ethylene glycol)) side chains: P‐3O‐3F, P‐3O‐5F, P‐3O‐7F, and the reference conjugated polymer P‐3O‐3H (PNDI2TEG‐2Tz^[^ ^44^ ^]^), along with the n‐type dopant, N‐DMBI. **Scheme** **1** presents the synthetic route to the naphthalenediimide monomers and polymers featuring polar glycol ether side chains terminated with fluorinated alkyl chains. Briefly, starting from commercial 2,2′‐(ethane‐1,2‐diylbis(oxy))diethanol **1**, tosylation of the dialcohol yielded compound **2**. This was then selectively reacted with fluorinated alkyl chains alcohol, which was subsequently used to convert to primary amino compounds via Gabriel reaction. Then, the target naphthalenediimide monomers were obtained by reacting the primary amino compounds with 2,6‐dibromonaphthalene‐1,4,5,8‐tetracarboxylic dianhydride. The details of synthesis and characterization are presented in the Supporting Information (Figures S1–S35 and Schemes S1–S6 (Supporting Information), including ^1^HNMR spectra, ^13^CNMR spectra, ^19^FNMR, HRMS spectra, synthetic routines and GPC spectra). The copolymers were synthesized from the dibromo‐NDI‐based monomer and distannyl‐alkoxybithiazole‐based monomer via a copper iodide‐assisted Stille coupling polycondensation reaction using Pd_2_(dba)_3_/P(o‐tol)_3_ as the catalyst/ligand system, and the detailed synthetic procedures are described in the Supporting Information. After polymerization, the copolymers were purified using a Soxhlet extractor and continuous extraction with hot methanol, followed by hexane and acetone to remove impurities and low‐molecular‐weight fractions. Finally, the copolymers with high molecular weight were extracted using chloroform. Then, the polymer was dissolved and precipitated into methanol to give the product polymers. The structures were characterized using proton nuclear magnetic resonance (^1^H NMR). The relative molecular weights were determined using gel permeation chromatography (GPC) against polystyrene standards with chloroform as the eluent. The resulting GPC traces and data are listed in Figure S29 (Supporting Information) and **Table** **1**, respectively.

**Table 1:** Optical properties, electrochemical properties, and energy levels of the NDI‐based copolymers.

Polymers	P‐3O‐3F	P‐3O‐5F	P‐3O‐7F	P‐3O‐3H ^a)^
*M*_n_ (g mol^−1^)	23 301	29 702	31 138	26 231
*M*_W_ (g mol^−1^)	55 059	67 556	88 057	29 666
PDI	2.36	2.27	2.82	1.13
*φ* ^red^_onset_ (V)	−0.75	−0.75	−0.78	−0.80
*E*_red_ ^b)^ (eV)	−4.35	−4.35	−4.32	−4.30
*E*_ox_ ^c)^ (eV)	−5.41	−5.41	−5.38	−5.36

^a)^ Data from the literature;^[^ ^44^ ^]^

^b)^ *E*_red_ = ‐(5.10 +*E* ^red^_onset_) eV;

^c)^ *E*_ox_ = ‐(5.10 +*E* ^ox^_onset_) eV.

We investigated the optical properties of P‐3O‐3F, P‐3O‐5F, and P‐3O‐7F in the dilute chloroform solutions and solid‐state thin films by ultraviolet‐visible‐near infrared (UV–vis–NIR) absorption spectroscopy. The resulting absorption spectra and those of the reference polymer P‐3O‐3H (PNDI2TEG‐2Tz) are illustrated in Figure S36 (Supporting Information). The corresponding absorption parameters are summarized in Table 1. All pristine polymers exhibited similar two characteristic neutral absorption bands in both solution and thin film states, indicating that the introduction of fluorinated alkyl chains terminating polar glycol ether side chains into the NDI unit has a negligible effect on the absorption properties of the polymers. The optical bandgaps of these three polymers are 1.06 eV, calculated from their thin film absorption onsets. Interestingly, we found that the ratio of high‐energy bands (300–600 nm, π–π* transition) to low‐energy bands (600–1200 nm, intramolecular charge transfer (ICT)) decreases after introducing fluorinated alkyl chains. This may be because fluorinated alkyl chains promote the backbone planarity, enhancing the intramolecular charge transfer transition.

We then used cyclic voltammetry (CV) to evaluate the electronic properties of these polymers. The resulting plots are presented in Figure S37 (Supporting Information), and the corresponding parameters are summarized in Table 1. All polymers exhibited two pronounced reversible reduction waves in an acetonitrile solution containing 0.1 m tetrabutylammonium hexafluorophosphate electrolyte during the CV measurement, indicating their n‐doping (reduction) characteristics due to the strong electron‐deficient nature of the NDI‐2Tz backbone. The reduction energy levels (*E*_red_) of these three polymers were calculated from the onset reduction potentials (φ^red^_onset_) using the equation *E*_red_ = ‐(5.10 + φ^red^_onset_) eV. The φ^red^_onset_ values for P‐3O‐3F, P‐3O‐5F, and P‐3O‐7F were −0.75, −0.75, and −0.78 V, respectively, which corresponded to the estimated reduction energy levels of −4.35, −4.35, and −4.32 eV. Based on the optical bandgap and reduction energy levels, the oxidation energy levels of P‐3O‐3F, P‐3O‐5F, and P‐3O‐7F were calculated to be −5.41, −5.41, and −5.38 eV, respectively. Given the similar reduction energy levels of these three polymers and the reference polymer P‐3O‐3H, with slight differences and measurement errors within the CV measurements, we can exclude the influence of the side chains on the energetics.

The deep‐lying reduction energy levels of these polymers are beneficial for achieving efficient n‐doping, and the n‐doping behavior of three polymers was investigated by UV–vis–NIR absorption spectroscopy and electron paramagnetic resonance (EPR) spectroscopy. 4‐(1,3‐Dimethyl‐2,3‐dihydro‐1H‐benzoimidazol‐2‐yl)phenyl)dimethylamine (N‐DMBI) was selected as the n‐type dopant because of its strong n‐doping ability, good solution processability, and good stability.

**Figure 2:**
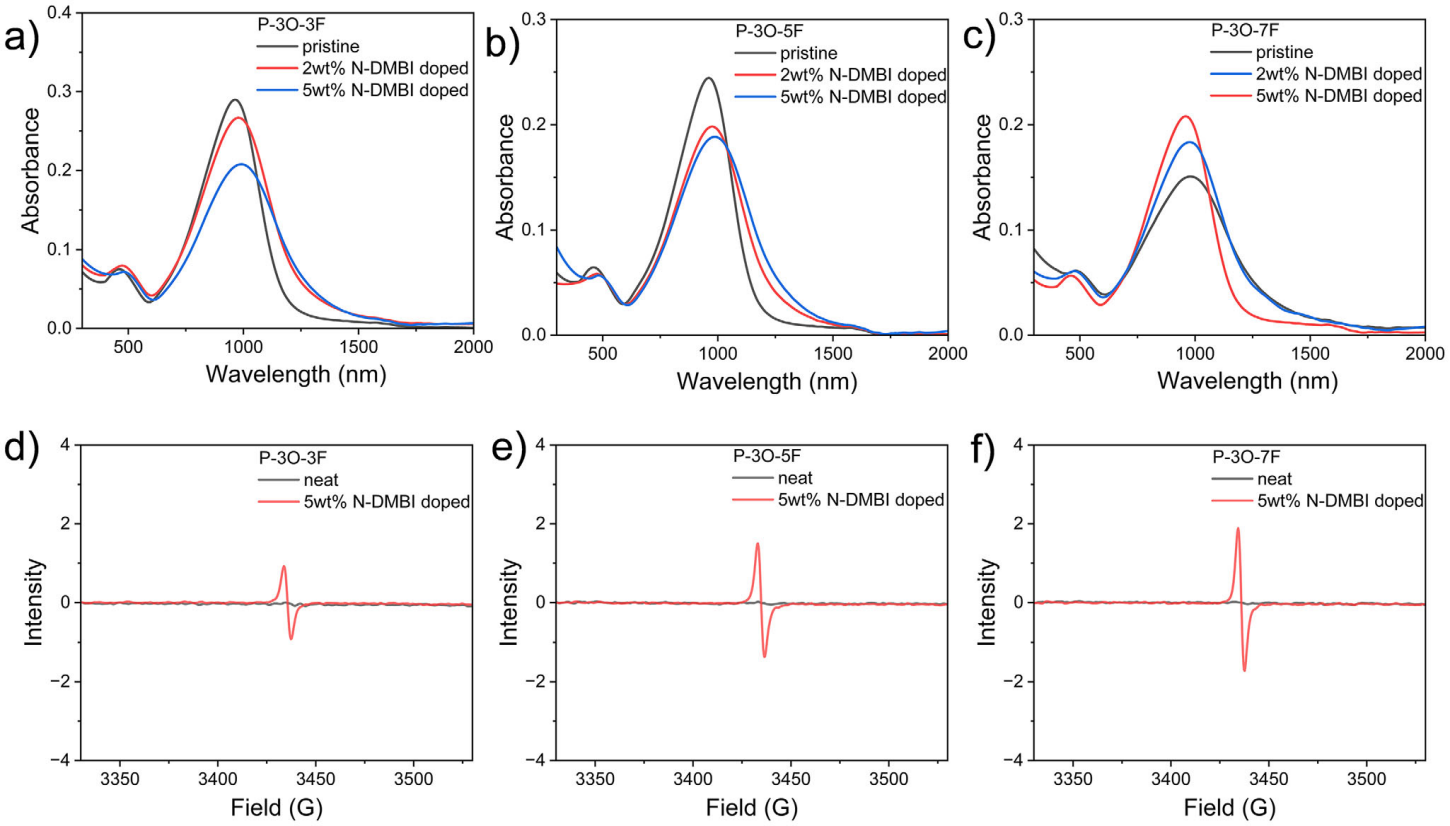
UV–vis–NIR thin film absorption spectra of the pristine and doped a) P‐3O‐3F, b) P‐3O‐5F, and c) P‐3O‐7F. EPR spectra of the pristine and doped d) P‐3O‐3F, e) P‐3O‐5F, and f) P‐3O‐7F.

**Figure** **2**a–c shows the UV–vis–NIR absorption spectra for pristine and doped copolymer thin films (the absorption spectra of reference polymer could be found in Figure S38, Supporting Information). No obvious absorption bands beyond 1200 nm for all pristine films were found. After molecular doping with N‐DMBI, all polymers' neutral ICT absorption bands gradually diminish as the dopant loading increases. In contrast, new substantial (bi)polaron absorption emerges in the near‐infrared region, indicating that all polymers were strongly n‐doped with N‐DMBI.

As illustrated in Figure 2d–f, all neat polymer films are electron spin resonance signals silent. Upon molecular doping, clear electron spin resonance signals were observed, attributed to the formation of numerous radical anions (polarons) in the doped polymer film. The extracted spin density for the doped P‐3O‐3H film reached a maximum of ≈8.59×10^19^ cm^−3^. While the calculated spin density of the doped P‐3O‐3F, P‐3O‐5F, and P‐3O‐7F films were slightly lower, ≈4.36×10^19^, 6.58×10^19^, and 6.55×10^19^ cm^−3^, respectively, at the same doping condition. Based on the spin density, the efficiency of polaron generation can be determined to be 26.1%, 36.1%, and 33.2%, respectively.

### 2.2 Thermoelectric and Charge Transport Measurements

**Figure 3:**
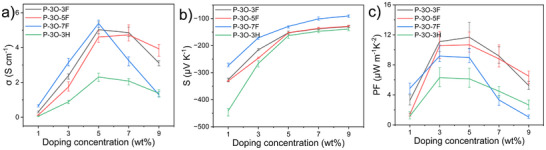
a) Electrical conductivities, b) Seebeck coefficients, and c) power factors recorded for P‐3O‐3F, P‐3O‐5F, and P‐3O‐7F at different doping ratios.

To evaluate the thermoelectric properties, we investigated the electrical conductivity (σ) and Seebeck coefficient (*S*) of doped P‐3O‐3F, P‐3O‐5F, and P‐3O‐7F as a function of the dopant weight ratio. **Figure** **3**a displays the σ values of the doped P‐3O‐3F, P‐3O‐5F, P‐3O‐7F, and the reference P‐3O‐3H thin films at different dopant loadings (see the details in the Supporting Information, device fabrication, and characterization section). All pristine polymer thin films exhibited insulating properties, and their σ values were below the measurement limit. After N‐DMBI doping, the *σ* values of all polymers gradually increased with dopant loading but then decreased at much higher dopant ratios. P‐3O‐3F, P‐3O‐5F, and P‐3O‐7F achieved maximum conductivities of 5.03, 4.73, and 5.39 S cm^−1^, respectively, double the optimal electrical conductivity of the reference P‐3O‐3H. Because these doped polymers had similar charge carrier densities, increased σ values resulted from enhanced charge mobility.

To gain insight into the *σ* difference for these polymers, the bulk charge mobility of the doped films was calculated as 0.72 cm^2^ V^−1^ s^−1^ for P‐3O‐3F, 0.45 cm^2^ V^−1^ s^−1^ for P‐3O‐5F, and 0.51 cm^2^ V^−1^ s^−1^ for P‐3O‐7F based on the measured σ and extracted spin density from the EPR measurements, according to the formula, μ = σ/ne. The calculated charge mobility value for P‐3O‐3H is 0.16 cm^2^ V^−1^ s^−1^. Therefore, the increase in *σ* values is due to the enhanced charge mobility, which may result from a higher degree of long‐range order in polymer packing due to the strong self‐organization of fluorinated alkyl chains.^[^ ^62^, ^63^ ^]^

We then measured the *S* values of the doped polymer films, and the resulting plots are presented in (Figure 3b). The *S* values of all the doped polymer thin films are negative, indicating that the dominant charge carriers are radical anions. The absolute *S* values of all polymers exhibited a consistent trend and gradually decreased as the dopant concentration increased, resulting from an increased radical anion density. For example, at 5wt% doping concentration, P‐3O‐3F, P‐3O‐5F, P‐3O‐7F, and P‐3O‐3H exhibited *S* values of −152.4, −152.2, −129, and −163 µV K^−1^, respectively. Based on *σ* mentioned above and *S* values, we calculated the power factor values of all the doped polymer films, summarized in Figure 3c. The maximum PFs of 11.68, 10.67, 9.18, and 6.29 µWm^−1^K^−2^ were obtained for P‐3O‐3F, P‐3O‐5F, P‐3O‐7F, and P‐3O‐3H. After introducing fluoro‐alkyl chains onto the polar OEG side chain, P‐3O‐3F, P‐3O‐5F, and P‐3O‐7F exhibited increased PFs due to enhanced conductivity.

### 2.3 Polymer Chain Packing and Film Morphology

We carried out the 2D grazing‐incidence wide‐angle X‐ray scattering (GIWAXS) and atomic force microscope (AFM) measurement for the pristine and doped polymer films to gain insight into the effects of the side chain variation and molecular doping on the molecular packing. As shown in Figure S39 (Supporting Information), all pristine P‐3O‐3F, P‐3O‐5F, and P‐3O‐7F films, and the reference polymers exhibited fibril‐textured morphologies. All the doped polymer film shows good surface morphology with few aggregates, revealing that all polymers had excellent dopant/polymer mixing due to the existing polar glycol ether segment in the side chain.

**Figure 4:**
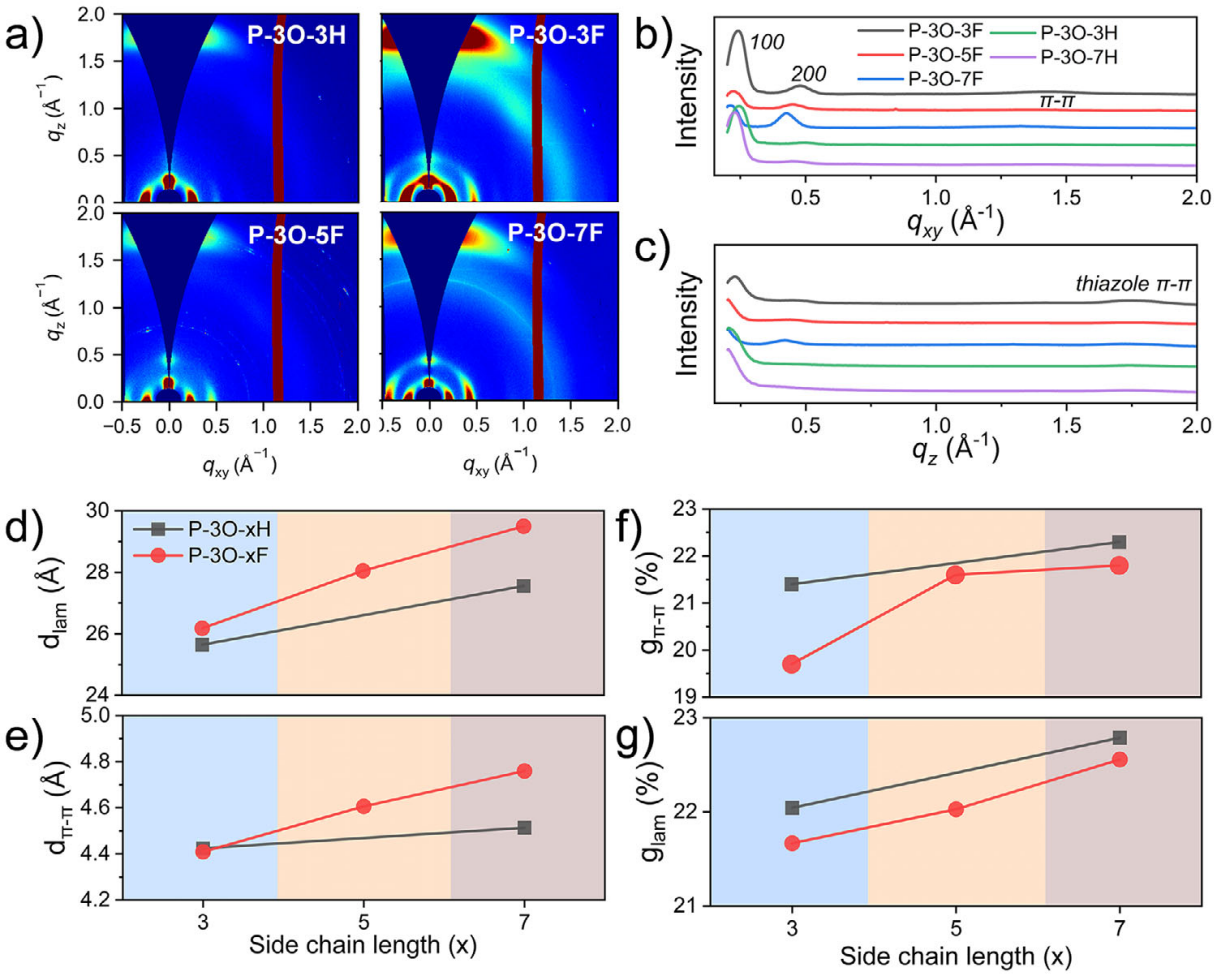
a) GIWAXS 2D patterns, and linecuts in the direction of b) *q_xy_* and c) *q_z_* of intrinsic P‐3O‐xF and P‐3O‐3H films, the 2D pattern of P‐3O‐7H and doped analogues could be found in Supporting information. Analysis of d) lamellar packing distance, e) π–π stacking space, paracrystalline disorder of f) lamellar packing and g) π–π stacking of the intrinsic polymer films.

**Figure** **4**a–c presents the 2D GIWAXS patterns and corresponding linecut analyses for pristine P‐3O‐3F, P‐3O‐5F, P‐3O‐7F polymers and reference polymer P‐3O‐3H, with additional data for P‐3O‐7H and doped films provided in Figure S40 (Supporting Information). All pristine polymer films exhibit mixed crystallographic orientation, as demonstrated by the simultaneous presence of (100) and (200) lamellar diffraction peaks in both out‐of‐plane (OOP, *q_z_* direction) and in‐plane (IP, *q_xy_* direction) orientations. Quantitative structural parameters derived from these measurements are summarized in Figure 4 and Table S1 (Supporting Information). The π–π stacking distances were determined from IP linecuts (*q_xy_* ≈1.6–1.8 Å⁻¹) since π‐aggregates aligned in the IP direction facilitate in‐plane charge transport, while lamellar spacings were similarly extracted from IP linecuts due to insufficient resolution of OOP (100) peaks in the *q_z_* direction. Systematic analysis reveals progressive structural expansion with side‐chain elongation (as shown in Figure 4d,e): lamellar spacing increases from ~26 Å (x = 3) to ~28 Å (x = 7) for P‐3O‐xH, and from ~26 to ~30 Å for P‐3O‐xF counterparts, accompanied by π–π stacking distance enlargement from ~4.4 to ~4.5 Å (H‐series) and ~4.4 to ~4.8 Å (F‐series). Concurrently, paracrystalline disorder for both lamellar and π–π stacking increases with chain length, suggesting diminishing structural order beyond optimal side‐chain dimensions (Figure 4f,g). Comparative evaluation of fluorinated versus non‐fluorinated analogs (P‐3O‐3F/P‐3O‐3H, for instance) demonstrates that fluorinated alkyl chains enhance crystalline order through reduced lamellar (Δ*g* = −0.3%) and π‐π (Δ*g* = −1.7%) disorder, attributable to their enhanced self‐organization capability.^[^ ^62^, ^63^ ^]^ This structural optimization correlates with improved charge transport properties. Remarkably, molecular doping with 5 wt% N‐DMBI preserves the pristine packing structure, indicating dopant localization within the polymer side‐chain matrix and structural robustness of the oligo(ethylene glycol) (OEG) side chains against doping‐induced perturbations.

### 2.4 Water Contact Angles

It is well‐known that fluoro‐containing polymers have hydrophobic features and are widely used as water‐repellent materials.^[^ ^64^, ^65^, ^66^ ^]^ Because of their superhydrophobic feature, fluorinated alkyl chains possess excellent water‐repellent properties, potentially functioning as molecular shields to keep water off and enabling applications in organic thermoelectric (OTE) materials with improved air stability. Here, we first investigated the water contact angles of thin films based on these polymers (Figure S41, Supporting Information). For comparison, the reference conjugated polymer PNDI2TEG‐2Tz (P‐3O‐3H) was also tested, which showed a water contact angle of 82.1°. After introducing fluorinated alkyl chains to the polar oligo (ethylene glycol) (OEG) side chains, P‐3O‐3F, P‐3O‐5F, and P‐3O‐7F showed increased water contact angles of 86.3°, 92.6°, and 98.0°, respectively, indicating gradually improving hydrophobic properties for NDI‐based polymers with increasing numbers of fluoro side chains. The improved hydrophobic properties will potentially enhance the water resistance of n‐doped thermoelectric polymers, benefiting their air stability.

### 2.5 Stability of Thermoelectric Polymers

The stability of n‐type doped thermoelectric polymers in air is crucial for their widespread applications. Currently, high electrical conductivity (σ>100 Scm^−1^) and thermoelectric performances (PF>100 µWm^−1^K^−2^) of n‐type doped polymers have been achieved.^[^ ^17^, ^28^ ^]^ However, they only work efficiently in an inert atmosphere. When exposed to ambient conditions, the electrical conductivity and thermoelectric performance of n‐type doped polymers decreased dramatically within a short time. This is caused by the quenching of the radical anions by H_2_O and O_2_ in ambient air.

**Figure 5:**
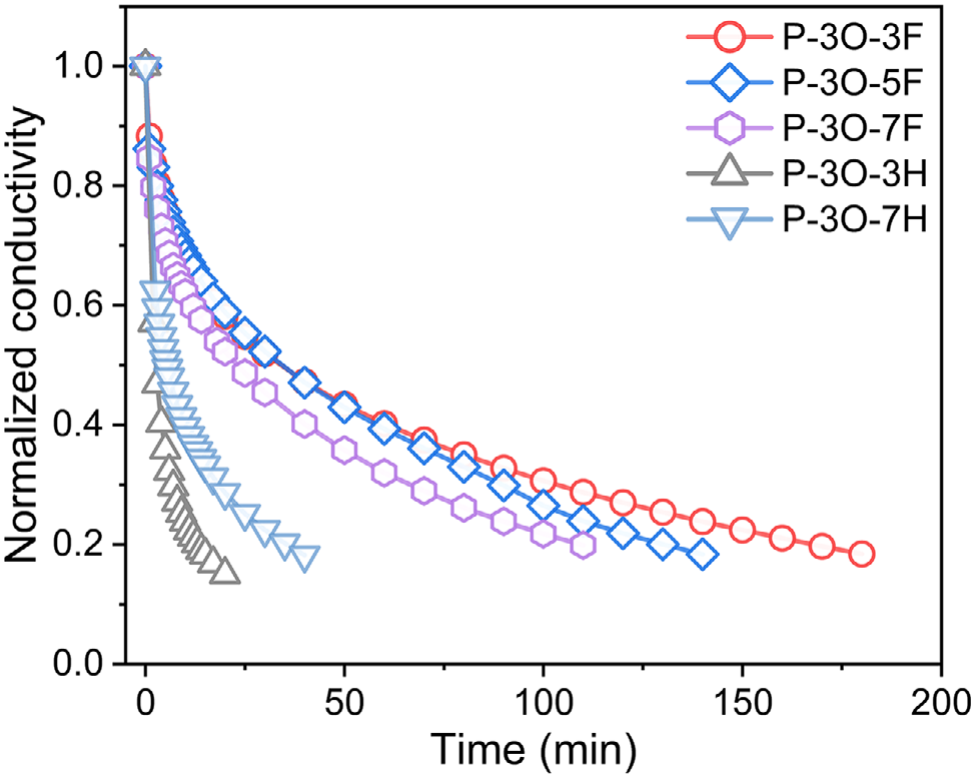
Electrical conductivity stability of all 5wt.% N‐DMBI doped polymer thin films in air.

**Figure** **5** presents the stability of doped polymer thin films in ambient air. The thicknesses of doped P‐3O‐3F, P‐3O‐5F, and P‐3O‐7F were 75, 40, and 40 nm, respectively. The electrical conductivity of doped P‐3O‐3H (thickness 50 nm) drops to 20% of its initial value within 15 min. After introducing fluoro side chains, n‐type doped P‐3O‐3F, P‐3O‐5F, and P‐3O‐7F exhibited significantly enhanced air stability and remained at 20% of their conductivities within 160, 130, and 100 min, respectively. To exclude the potential of the ending group on the OEG side chain effect, we also synthesized conjugated polymer P‐3O‐7H (Supporting Information), which has the same backbone but a polar OEG side chain with an alkyl chain ending group. P‐3O‐7H shows optimal electrical conductivity of 5.13 S cm^−1^, negative Seebeck coefficient, and maximum PF of 9.8 µWm^−1^K^−2^ after molecular doping with N‐DMBI (see Figure S42, Supporting Information). The conductivity of P‐3O‐7H drops to 20% of its initial value within 35 min, demonstrating that the higher air stability of n‐type doped polymer films of P‐3O‐3F, P‐3O‐5F, and P‐3O‐7F is due to the introduction of the fluorinated alkyl chain end group. Therefore, introducing the fluorinated alkyl chain as the OEG side chain end group acts as a molecular shield, protecting against water and oxygen ingress, leading to enhanced stability of doped polymer thin films in ambient air.

## 3 Conclusion

We have designed and synthesized three n‐type conjugated polymers featuring a naphthalenediimide and dialkoxybithiazole backbone. These polymers include polar OEG side chains with varying lengths of fluorinated alkyl chain end groups. We characterized and evaluated all the polymers as thermoelectric materials. After molecular doping with N‐DMBI, the doped films of the conjugated polymers modified with fluorinated alkyl side chains demonstrated higher electrical conductivity, resulting in an improved power factor and better air stability compared to the unmodified polymers that share the same conjugated backbone. The enhanced air stability of the n‐type doped polymers with fluorinated alkyl side chains can be attributed to the excellent water‐repellent properties of these fluorinated chains, which effectively act as a protective barrier against moisture. Our research provides valuable insights into material design for the future development of air‐stable n‐type organic thermoelectrics.

## Conflict of Interest

The authors declare no conflict of interest.

## Supporting information



Supporting Information

## Data Availability

The data that support the findings of this study are available from the corresponding author upon reasonable request.
