# Supplementary material for: Fluorinated Alkyl Chains Terminated Polar Glycol Ether Side Chain for N‐Type Organic Thermoelectrics with Enhanced Performance and Air Stability
Source: Adv Sci (Weinh). 2025 Apr 7;12(25):2500571. doi: 10.1002/advs.202500571 (PMC12224962; doi:10.1002/advs.202500571)
Supplement: Supplementary file 1 — Supporting Information [file ADVS-12-2500571-s001.docx]

Supporting Information

Fluorinated Alkyl Chains Terminated Polar Glycol Ether Side Chain Engineering Enhancing Stability and Performance of N-type Organic Thermoelectrics

Gang Ye,^[a]^ Yazhuo Kuang,^[b,c]^ Mingyu Ma,^[b,c]^ Xiantao Peng,^[b,c]^ and Jian Liu*^[b,c]^

[a] Key Laboratory for the Green Preparation and Application of Functional Materials, Hubei Key Laboratory of Polymer Materials, School of Materials Science and Engineering, Hubei University, Youyi Road 368, Wuhan 430062, P. R. China.

[b] State Key Laboratory of Polymer Science and Technology, Changchun Institute of Applied Chemistry, Chinese Academy of Sciences, Changchun 130022, P. R. China.

[c] School of Applied Chemistry and Engineering, University of Science and Technology of China, Hefei 230026, P. R. China.

#### Materials synthesis and characterization

**Reagents**: All reagents and solvents were commercial and were used as received. 4,9-dibromoisochromeno[6,5,4-def]isochromene-1,3,6,8-tetraone was purchased from Derthon. 4,4'-bis(octyloxy)-2,2'-bis(trimethylstannyl)-5,5'-bithiazole (2Tz) was purchased from SunaTech Inc.

**Characterization**: ^1^HNMR and ^13^CNMR spectra of organic molecules were performed on a Bruker AV-500 spectrometer at 25 ℃, using tetramethylsilane (TMS) as an internal standard. NMR shifts are reported in ppm, relative to the residual protonated solvent signals of CDCl_3_ (δ=7.26 ppm) or at the carbon absorption in CDCl_3_ (δ=77.23 ppm). Multiplicities are denoted as: singlet (s), doublet (d), triplet (t) and multiplet (m). GPC measurements were done on a Waters 1515 room temperature GPC/SEC system at 30 °C vs polystyrene standards using chloroform (CHCl_3_) as eluent.

**Cyclic Voltammetry (CV)**: Cyclic voltammetry (CV) measurements in organic solution were carried out with a CHI760 Evoltammetric potentiostat in a three-electrode configuration where the working electrode was glass carbon electrode, the counter electrode was a platinum wire, and the pseudo-reference was an Ag/AgCl wire that was calibrated against ferrocene (Fc/Fc^+^). Cyclic voltammograms for NDI-Based polymers film deposited on the glass carbon working electrode in CH_3_CN solution containing Bu_4_NPF_6_ (0.1 molL^-1^) electrolyte at a scanning rate of 100 mVs^-1^.

**Device fabrication**: Borosilicate glass/Si substrates were cleaned successively with a surfactant solution, water, acetone: water = 1:1 solution, and isopropanol in an ultrasonic cleaner for 20 minutes and then dried in a drying oven. Then, the clean borosilicate glass substrates were treated with a vacuum plasma cleaner (VR-RS6) for 3 minutes. The neat polymer solution was prepared, stirred overnight, and sonicated for 20 minutes before spin-coating. Polymer and dopant solutions were mixed before spin-coating. The polymer films were prepared by spin-coating a solution (5 mg/mL in hexafluoroisopropanol) at room temperature. The processed polymer films were annealed at 110 ℃ for 30 minutes and then 150 ℃ for 30 minutes. The conductivity of the doped polymer-activated films was tested using a four-point probe method in a glovebox with a nitrogen atmosphere. KLA Tencor P7 and BRUKER ContourGT were used to measure the film thickness.

**Water Contact angle measurement:** contact angle measurements were performed by Kruss DSA100.

**Electrical** **conductivity and Seebeck coefficient measurement**:

The electrical conductivity (σ) was measured in an N_2_ filled glovebox by a Keithley 2450 controlled by self-coded Labview. The parallel-shaped electrodes (2 nm Cr and 40 nm Au) were deposited at the surface of the glass before the film spin-coated to form the bottom contact. The four probes electrical conductivity was calculated as σ=(I/V)×l/(w×d), for which the channel length (l) is 0.5 mm, channel width (w) is 3.0 mm, and the film thickness (d) is nearly 30-50 nm.

**Seebeck Coefficient Measurement**: The Seebeck coefficient(s) was also measured in an inert N_2_ atmosphere. A set of Peltier was employed to serve as a temperature gradient. During measurement, the temperature variation and voltage difference were monitored by DAQ970A and DAQ973A respectively.

**Electron paramagnetic resonance (EPR):** Electron paramagnetic resonance (EPR) spectra were measured through a Bruker EMXnano EPR spectrometer (The microwave power was 1.0 mW, the modulation amplitude was 3.0 G, the microwave attenuation was 40.0 dB). The Xenon software was used to collect data and calculate spin density. The samples with all batches of NDI-based polymers were prepared as film fabrication.

**Atomic Force Microscopy (AFM):** The AFM measurement was performed on a Bruker Dimension ICON AFM in peak force mode under ambient conditions. The samples with all batches of NDI-based polymers were prepared as film fabrication.

**Grazing Incidence Wide Angle Scattering (GIWAXS)**: The GIWAXS data were obtained at 1W1A Diffuse X-ray Scattering Station, Beijing Synchrotron Radiation Facility (BSRF-1W1A). The incidence angle was performed at 0.3°. The NDI-based polymers were deposited on silicone. The reshaped GIWAXS patterns, considering the inaccessible part in reciprocal space (wedge-shaped corrected patterns), are presented as a function of the vertical and parallel scattering vectors q_z_ and q_r_. The scattering vector coordinates for the GIWAXS geometry are given by:

$q=\left\{ \begin{aligned} q_{x}=\frac{2\pi}{\lambda}\left( cos(2\theta_{f})\cos\left( \alpha_{f} \right)-cos(\alpha_{i}) \right) \\ q_{y}=\frac{2\pi}{\lambda}\left( \sin\left( 2\theta_{f} \right)\cos\left( \alpha_{f} \right) \right) \\ q_{z}=\frac{2\pi}{\lambda}\left( \sin\left( \alpha_{i} \right)+sin(\alpha_{f}) \right) \end{aligned} \right.$ (S1)

where $2\theta_{f}$ is the scattering angle in the horizontal direction and $\alpha_{f}$ is the exit angle in the vertical direction. The parallel component of the scattering vector is thus calculated as $q_{r}=\sqrt{q_{x}^{2}+q_{y}^{2}}$.

**Synthetic procedures**

Scheme S1. Synthetic route to NDI-based monomer **NDI-3O-3F**.

(ethane-1,2-diylbis(oxy))bis(ethane-2,1-diyl) bis(4-methylbenzenesulfonate) **(2)**

To a stirred solution of 2,2'-(ethane-1,2-diylbis(oxy))diethanol (30 g, 200 mmol) in 80 mL of THF at 0 ^o^C, a solution of NaOH (95.3 g) dissolved in 130 mL of water was added, the resulting mixture was stirring for 2 hours at 0 ^o^C. Then, dropwise, a solution of p-toluenesulfonyl chloride (95.3 g, 500 mmol) in THF (80 mL) was added. The reaction mixture was allowed to warm to room temperature and stirred overnight. After workup, the reaction mixture was washed with brine for three times, dried over anhydrous Na_2_SO_4_, filtered, concentrated, and dried under vacuum to afford the crude product as a white solid (89 g, 97%). The crude product was used in the next reaction without further purification.

^1^HNMR (400 MHz, CDCl_3_): δ (ppm) 7.78 (d, J=8.2, 4H), 7.33 (d, J=8.1, 4H), 4.13 (m, 4H), 3.64 (m, 4H), 3.51 (s, 4H), 2.43 (s, 6H).

2-(2-(2-(2,2,2-trifluoroethoxy)ethoxy)ethoxy)ethyl 4-methylbenzenesulfonate **(3)**

In flask one, sodium hydride (60 % in mineral oil, 2.4 g, 10 mmol) was added under nitrogen. Then anhydrous THF (5 mL) was added and the mixture was cooled in an ice bath. 2,2,2-trifluoroethanol (500 mg, 5 mmol) was added dropwise while keeping the flask under ice bath. After dropping, the solution was stirred for 15 minutes before removing the ice bath. Then, the reaction mixture was stirred at room temperature for 1 hour. In another flame dry flask, (ethane-1,2-diylbis(oxy))bis(ethane-2,1-diyl) bis(4-methylbenzenesulfonate) (10 g, 21.83 mmol) was dissolved in THF (50 mL) The reaction mixture in last flask was transfer and added dropwise by a syringe at 0 ^o^C and kept stirring for 1h. The solution was warm to room temperature and stirred overnight before it was quenched with water. After workup, the reaction mixture was washed with brine three times, dried over anhydrous Na2SO4, filtered, and concentrated. The crude product was purified by silica gel column chromatography with petroleum ether/ethyl acetate (1:4) to afford the target compound as a colorless oil (1.6 g, 83%).

^1^HNMR (400 MHz, CDCl_3_): δ (ppm) 7.79 (d, J=7.8 Hz, 2H), 7.34 (d, J=7.8 Hz, 2H), 4.16 (t, J = 4.8 Hz, 2H), 3.77 (m, 2H), 3.70 (m, 2H), 3.64 (m, 2H), 3.59 (m, 2H), 3.54-3.43 (m, 4H), 2.45 (s,3H).

^19^FNMR (100 MHz, CDCl_3_): δ (ppm) -74.27.

Figure S1: The ^1^HNMR spectra of 2-(2-(2-(2,2,2-trifluoroethoxy)ethoxy)ethoxy)ethyl 4-methylbenzenesulfonate.

Figure S2: The ^19^FNMR spectra of 2-(2-(2-(2,2,2-trifluoroethoxy)ethoxy)ethoxy)ethyl 4-methylbenzenesulfonate.

2-(2-(2-(2-(2,2,2-trifluoroethoxy)ethoxy)ethoxy)ethyl)isoindoline-1,3-dione **(4)**

2-(2-(2-(2,2,2-trifluoroethoxy)ethoxy)ethoxy)ethyl-4-methylbenzenesulfonate (1.6 g, 4.15 mmol) and potassium phthalimide (926 mg, 5 mmol) were dissolved in DMF (5 mL). The reaction mixture was stirred at 100 ^o^C overnight. After cooling to room temperature, deionized water was added and the mixture was extracted by ethyl acetate, the organic phase was collected, dried over anhydrous Na_2_SO_4_, filtered, concentrated. The crude product was purified by silica gel column chromatography with petroleum ether/ethyl acetate (1:2) as the eluent to afford target compound as a colorless oil (1.4 g, 96%).

^1^HNMR (400 MHz, CDCl_3_): δ (ppm) 7.77 (dd, J=5.4, 3.1, 2H), 7.64 (dd, J=5.5, 3.1, 2H), 3.868-3.744 (m, 4H), 3.696-3.618 (m, 4H), 3.595-3.497 (m, 6H).

^19^FNMR (400 MHz, CDCl_3_): δ (ppm) -74.30.

Figure S3: The ^1^HNMR spectra of 2-(2-(2-(2-(2,2,2-trifluoroethoxy)ethoxy)ethoxy) ethyl)isoindoline-1,3-dione.

Figure S4: The ^19^FNMR spectra of 2-(2-(2-(2-(2,2,2-trifluoroethoxy)ethoxy)ethoxy) ethyl)isoindoline-1,3-dione.

2-(2-(2-(2,2,2-trifluoroethoxy)ethoxy)ethoxy)ethanamine **(5)**

2-(2-(2-(2-(2,2,2-trifluoroethoxy)ethoxy)ethoxy)ethyl)isoindoline-1,3-dione (1.44 g, 4 mmol), hydrazine hydrate (0.58 mL, 12 mmol) and 50 mL ethanol were stirred at 95 ^o^C for 3 hours. Then, 2 mL con. HCl was added and refluxed for another hour. After cooling to room temperature, the ethanol was evaporated by rotary evaporation, the residue was diluted with CH_2_Cl_2_ and washed with 10% NaOH. Aqueous layers were combined and extracted with CH_2_Cl_2_. The combined organic layers were washed with brine and dried over Na_2_SO_4_. The removal of CH_2_Cl_2_ afforded yellow oil as amine product which was used without further purification.

4,9-dibromo-2,7-bis(2-(2-(2-(2,2,2-trifluoroethoxy)ethoxy)ethoxy)ethyl)benzo[lmn][3,8]phenanthroline-1,3,6,8(2H,7H)-tetraone **(NDI-3O-3F)**

2-(2-(2-(2,2,2-trifluoroethoxy)ethoxy)ethoxy)ethanamine (560 mg, 2.4 mmol, 4 eq) was added to a suspension of 2,6-dibromo-1,4,5,8-naphthalene tetracarboxylic diimide (213 mg, 0.5 mmol, 1 eq) in 10 mL of glacial acetic acid and heated to 140 ^o^C for 1 h. The reaction mixture was then cooled to room temperature and added to water, then extracted with CH_2_Cl_2_. The organic phase was washed with brine, dried over anhydrous Na_2_SO_4_ and removed solvent by rotary evaporator. The crude product was purified by silica gel column chromatography with CH_2_Cl_2_/acetone as the eluent afforded target compound as a light red solid (230 mg, 54%).

^1^HNMR (400 MHz, CDCl_3_): δ (ppm) 8.98 (s, 2H), 4.47 (t, J= 5.8, 4H), 3.912-3.816 (m, 8H), 3.747-3.675 (m, 8H), 3.654-3.59 (m, 8H).

^13^CNMR (100 MHz, CDCl_3_): δ (ppm) 160.87, 160.78, 139.06, 128.38, 127.80, 125.34, 124.12, 71.93, 70.75, 70.62, 70.11, 69.13, 68.73 (q, J=33.8), 40.03.

^19^FNMR (400 MHz, CDCl_3_): δ (ppm) -74.30.

HRMS Calcd. for C_30_H_31_Br_2_F_6_N_2_O_10_ [M+H]^+^ m/z: 853.02292, found: 853.02734. Calcd. for C_30_H_30_Br_2_F_6_N_2_O_10_Na_1_ [M+Na]^+^ m/z: 875.00486, found: 875.00096.

Figure S5: The ^1^HNMR spectra of 4,9-dibromo-2,7-bis(2-(2-(2-(2,2,2-trifluoroethoxy) ethoxy)ethoxy)ethyl)benzo[lmn][3,8]phenanthroline-1,3,6,8(2H,7H)-tetraone.

Figure S6: The ^13^CNMR spectra of 4,9-dibromo-2,7-bis(2-(2-(2-(2,2,2-trifluoroethoxy) ethoxy)ethoxy)ethyl)benzo[lmn][3,8]phenanthroline-1,3,6,8(2H,7H)-tetraone.

Figure S7: The ^19^FNMR spectra of 4,9-dibromo-2,7-bis(2-(2-(2-(2,2,2-trifluoroethoxy) ethoxy)ethoxy)ethyl)benzo[lmn][3,8]phenanthroline-1,3,6,8(2H,7H)-tetraone.

Figure S8: The HRMS spectra of 4,9-dibromo-2,7-bis(2-(2-(2-(2,2,2-trifluoroethoxy) ethoxy)ethoxy)ethyl)benzo[lmn][3,8]phenanthroline-1,3,6,8(2H,7H)-tetraone.

Scheme S2. Synthetic route to NDI based monomer **NDI-3O-5F**.

2-(2-(2-(2,2,3,3,3-pentafluoropropoxy)ethoxy)ethoxy)ethyl4-methylbenzenesulfonate (6)

In one flask, sodium hydride (60 % in mineral oil, 800 mg, 20 mmol) was added under nitrogen. Then anhydrous THF (10 mL) was added and the mixture was cooled in an ice bath. 2,2,3,3,3-pentafluoropropan-1-ol (1500 mg, 10 mmol) was added dropwise while keeping the flask under ice bath. After dropping, the solution was stirred for 30 min before the ice bath was removed. Then reaction solution was stirred at room temperature for 2.5 h. In another flame dry flask, (ethane-1,2-diylbis(oxy))bis(ethane-2,1-diyl) bis(4-methylbenzenesulfonate) (22.9 g, 50 mmol) was dissolved in THF (50 mL) The reaction mixture in last flask was transfer and added dropwise by a syringe at 0 ^o^C and kept stirring for 1h. The solution was warm to room temperature and stirred overnight before it was quenched with water. After workup, the reaction mixture was washed with brine for three times, dried over anhydrous Na_2_SO_4_, filtered, concentrated. The crude product was purified by silica gel column chromatography with petroleum ether/ethyl acetate (3:1) as the eluent to afford target compound as a colorless oil (3.31 g, 76%).

^1^HNMR (500 MHz, CDCl_3_): δ (ppm) 7.77 (d, J = 8.3 Hz, 2H), 7.32 (d, J = 8.1 Hz, 2H), 4.13(t, J = 4.8 Hz, 2H), 4.03-3.91 (m, 2H), 3.77-3.71 (m, 2H), 3.69-3.60 (m, 4H), 3.59-3.52 (m, 4H), 2.45 (s, 3H).

^13^CNMR (125 MHz, CDCl_3_): δ (ppm) 144.86, 132.97, 129.85, 129.81, 127.93, 122.13, 120.13, 119.85, 119.57, 117.86, 117.58, 117.30, 115.30, 115.01, 113.57, 113.28, 112.98, 112.69, 111.25, 110.95, 72.16, 70.70, 70.61, 70.59, 69.23, 68.66, 67.84 (t, J = 26.0 Hz), 21.53.

^19^FNMR (400 MHz, CDCl_3_): δ (ppm) -83.68, -123.62.

Figure S9: The ^1^HNMR spectra of 2-(2-(2-(2,2,3,3,3-pentafluoropropoxy)ethoxy) ethoxy)ethyl 4-methylbenzenesulfonate.

Figure S10: The ^13^CNMR spectra of 2-(2-(2-(2,2,3,3,3-pentafluoropropoxy)ethoxy) ethoxy)ethyl 4-methylbenzenesulfonate.

Figure S11: The ^19^FNMR spectra of 2-(2-(2-(2,2,3,3,3-pentafluoropropoxy)ethoxy) ethoxy)ethyl 4-methylbenzenesulfonate.

2-(2-(2-(2-(2,2,3,3,3-pentafluoropropoxy)ethoxy)ethoxy)ethyl)isoindoline-1,3-dione (**7**)

3-(2-(2-(2,2,3,3,4,4,4-heptafluorobutoxy)ethoxy)ethoxy)ethyl-4-methylbenzenesulfonate (3.3 g, 7.56 mmol) and potassium phthalimide (1.68 g, 9.10 mmol) were dissolved in DMF (10 mL). The reaction mixture was stirred at 100 ^o^C overnight. After cooling to room temperature, deionized water was added and the mixture was extracted by ethyl acetate, the organic phase was collected, dried over anhydrous Na_2_SO_4_, filtered, concentrated. The crude product was purified by silica gel column chromatography with petroleum ether/ethyl acetate (3:1) as the eluent to afford target compound as a colorless oil (2.25 g, 60%).

^1^HNMR (500 MHz, CDCl_3_): δ (ppm) 7.84 (dd, J = 5.4, 3.0 Hz, 2H), 7.72 (dd, J = 5.4, 3.0 Hz, 2H) 4.06-3.88 (m, 4H), 3.86-3.56 (m, 10H).

^13^CNMR (126 MHz, CDCl_3_): δ (ppm) 168.27, 133.95, 132.16, 123.24, 120.15, 119.87, 119.59, 117.87, 117.60, 117.32, 115.32, 115.03, 113.30, 113.00, 111.27, 110.97, 72.20, 70.72, 70.67, 70.11, 68.12, 67.96, 67.92, 67.71.

^19^FNMR (400 MHz, CDCl_3_): δ (ppm) -83.64, -123.58.

Figure S12: The ^1^HNMR spectra of 2-(2-(2-(2-(2,2,3,3,3-pentafluoropropoxy)ethoxy) ethoxy)ethyl)isoindoline-1,3-dione.

Figure S13: The ^13^CNMR spectra of 2-(2-(2-(2,2,3,3,3-pentafluoropropoxy)ethoxy) ethoxy)ethyl 4-methylbenzenesulfonate.

Figure S14: The ^19^FNMR spectra of 2-(2-(2-(2,2,3,3,3-pentafluoropropoxy)ethoxy) ethoxy)ethyl 4-methylbenzenesulfonate.

2-(2-(2-(2,2,3,3,3-pentafluoropropoxy)ethoxy)ethoxy)ethanamine (**8**)

2-(2-(2-(2-(2,2,3,3,4,4,4-heptafluorobutoxy)ethoxy)ethoxy)ethyl)isoindoline-1,3-dione (2.25 g, 5.47 mmol), hydrazine hydrate (0.93 mL, 19.15 mmol) and ethanol (150 mL) were stirred at 95 ^o^C for overnight. After cooling to room temperature, the ethanol was evaporated by rotary evaporation, the residue was diluted with CH_2_Cl_2_ and washed with 10% NaOH. Aqueous layers were combined and extracted with CH_2_Cl_2_. The combined organic layers were washed with brine and dried over Na2SO4. The removal of CH_2_Cl_2_ afforded yellow oil (1.55 g) as amine product which was used without further purification.

4,9-dibromo-2,7-bis(2-(2-(2-(2,2,3,3,3-pentafluoropropoxy)ethoxy)ethoxy)ethyl)benzo[lmn][3,8]phenanthroline-1,3,6,8(2H,7H)-tetraone (**NDI-3O-5F**)

1. (2-(2-(2,2,3,3,3-pentafluoropropoxy)ethoxy)ethoxy)ethanamine (1500 mg, 5.5 mmol, 4 eq) was added to a suspension of 2,6-dibromo-1,4,5,8-naphthalene tetracarboxylic diimide (588 mg, 1.38 mmol, 1 eq) in 20 mL of glacial acetic acid and heated to 140 ^o^C for 1 h. The reaction mixture was then cooled to room temperature and added to water, then extracted with CH_2_Cl_2_. The organic phase was washed with brine, dried over anhydrous Na_2_SO_4_ and removed solvent by rotary evaporator. The crude product was purified by silica gel column chromatography with CH_2_Cl_2_/acetone as the eluent afforded target compound as a yellow solid (640 mg, 49%).

^1^HNMR (400 MHz, CDCl_3_): δ (ppm) 8.99 (s, 2H), 4.47 (t, J = 5.8 Hz, 4H), 4.00-3.91 (m, 4H), 3.88-3.82 (m, 4H), 3.74-3.67 (m, 8H), 3.64-3.59 (m, 8H).

^13^CNMR (125 MHz, CDCl_3_): δ (ppm) 160.84, 160.75, 139.05, 128.37, 127.78, 125.33, 124.11, 122.10, 120.11, 119.83, 119.55, 117.83, 117.55, 117.28, 115.29, 115.00, 113.26, 112.97, 111.23, 110.94, 72.21, 70.77, 70.63, 70.10, 68.10, 67.90, 67.69, 67.62, 40.02.

^19^FNMR (400 MHz, CDCl_3_): δ (ppm) -83.62，-123.55.

HRMS Calcd. for C_32_H_31_Br_2_F_10_N_2_O_10_ [M+H]^+^: 953.01653, found: 953.01415, Calcd. for C_32_H_30_Br_2_F_10_N_2_O_10_Na_1_ [M+Na]^+^: 974.99848, found: 974.99537.

Figure S15: The ^1^HNMR spectra of 4,9-dibromo-2,7-bis(2-(2-(2-(2,2,3,3,3- pentafluoropropoxy)ethoxy)ethoxy)ethyl)benzo[lmn][3,8]phenanthroline-1,3,6,8(2H,7H)-tetraone.

Figure S16: The ^13^CNMR spectra of 4,9-dibromo-2,7-bis(2-(2-(2-(2,2,3,3,3-penta fluoropropoxy)ethoxy)ethoxy)ethyl)benzo[lmn][3,8]phenanthroline-1,3,6,8(2H,7H)-tetraone.

Figure S17: The ^19^FNMR spectra of 4,9-dibromo-2,7-bis(2-(2-(2-(2,2,3,3,3-penta fluoropropoxy)ethoxy)ethoxy)ethyl)benzo[lmn][3,8]phenanthroline-1,3,6,8(2H,7H)-tetraone.

Figure S18: The HRMS spectra of 4,9-dibromo-2,7-bis(2-(2-(2-(2,2,3,3,3-penta fluoropropoxy)ethoxy)ethoxy)ethyl)benzo[lmn][3,8]phenanthroline-1,3,6,8(2H,7H)-tetraone.

Scheme S3. Synthetic route to NDI based monomer **NDI-3O-7F**.

3-(2-(2-(2,2,3,3,4,4,4-heptafluorobutoxy)ethoxy)ethoxy)ethyl-4-methylbenzenesulfonate (**9**)

In one flask, sodium hydride (60 % in mineral oil, 800 mg, 20 mmol) was added under nitrogen. Then anhydrous THF (10 mL) was added and the mixture was cooled in an ice bath. 2,2,3,3,4,4,4-heptafluorobutan-1-ol (2000 mg, 10 mmol) was added dropwise while keeping the flask under ice bath. After dropping, the solution was stirred for 30 min before the ice bath was removed. Then reaction solution was stirred at room temperature for 1.5 h. In another flame dry flask, (ethane-1,2-diylbis(oxy))bis(ethane-2,1-diyl) bis(4-methylbenzenesulfonate) (22.9 g, 50 mmol) was dissolved in THF (50 mL) The reaction mixture in last flask was transfer and added dropwise by a syringe at 0 ^o^C and kept stirring for 1h. The solution was warm to room temperature and stirred overnight before it was quenched with water. After workup, the reaction mixture was washed with brine for three times, dried over anhydrous Na_2_SO_4_, filtered, concentrated. The crude product was purified by silica gel column chromatography with petroleum ether/ethyl acetate (2:1) as the eluent to afford target compound as a colorless oil (3.1 g, 84%).

^1^HNMR (400 MHz, CDCl_3_): δ (ppm) 7.80 (d, J=8.3, 2H), 7.34 (d, J=8.1, 2H), 4.16 (t, J = 4.8 Hz, 2H), 4.06-3.96 (m, 2H), 3.78-3.73 (m, 2H), 3.73-3.67 (m, 2H), 3.65-3.61 (m, 2H), 3.59 (s, 4H), 2.45 (s, 3H).

^13^CNMR (100 MHz, CDCl_3_): δ (ppm) 171.22, 144.85, 132.96, 129.80, 127.90, 72.21, 70.66, 70.57, 69.23, 68.62, 67.91, 60.33, 21.46, 14.07.

^19^FNMR (400 MHz, CDCl_3_): δ (ppm) -80.97 (t, J= 9.7 Hz), -120.74 — -120.79 (m), -127.80 (s).

Figure S19: The ^1^HNMR spectra of 3-(2-(2-(2,2,3,3,4,4,4-heptafluorobutoxy)ethoxy) ethoxy)ethyl-4-methylbenzenesulfonate.

Figure S20: The ^13^CNMR spectra of 3-(2-(2-(2,2,3,3,4,4,4-heptafluorobutoxy)ethoxy) ethoxy)ethyl-4-methylbenzenesulfonate.

Figure S21: The ^19^FNMR spectra of 3-(2-(2-(2,2,3,3,4,4,4-heptafluorobutoxy)ethoxy) ethoxy)ethyl-4-methylbenzenesulfonate.

2-(2-(2-(2-(2,2,3,3,4,4,4-heptafluorobutoxy)ethoxy)ethoxy)ethyl)isoindoline-1,3-dione (**10**)

3-(2-(2-(2,2,3,3,4,4,4-heptafluorobutoxy)ethoxy)ethoxy)ethyl-4-methylbenzenesulfonate (3.1 g, 6.35 mmol) and potassium phthalimide (1.42 g, 7.65 mmol) were dissolved in DMF (10 mL). The reaction mixture was stirred at 110 ^o^C overnight. After cooling to room temperature, deionized water was added and the mixture was extracted by ethyl acetate, the organic phase was collected, dried over anhydrous Na_2_SO_4_, filtered, concentrated. The crude product was purified by silica gel column chromatography with petroleum ether/ethyl acetate (3:1) as the eluent to afford target compound as a colorless oil (1.99 g, 68%).

^1^HNMR (400 MHz, CDCl_3_): δ (ppm) 7.80 (d, J=8.3, 2H), 7.34 (d, J=8.1, 2H), 4.16 (t, J = 4.8 Hz, 2H), 4.06-3.96 (m, 2H), 3.78-3.73 (m, 2H), 3.73-3.67 (m, 2H), 3.65-3.61 (m, 2H), 3.59 (s, 4H), 2.45 (s, 3H).

^13^CNMR (100 MHz, CDCl_3_): δ (ppm) 171.22, 144.85, 132.96, 129.80, 127.90, 72.21, 70.66, 70.57, 69.23, 68.62, 67.91, 60.33, 21.46, 14.07.

^19^FNMR (400 MHz, CDCl_3_): δ (ppm) -80.98 (t, J= 9.7 Hz), -120.76 — -120.80 (m), -127.82.

Figure S22: The ^1^HNMR spectra of 2-(2-(2-(2-(2,2,3,3,4,4,4-heptafluorobutoxy) ethoxy)ethoxy)ethyl)isoindoline-1,3-dione.

Figure S23: The ^13^CNMR spectra of 2-(2-(2-(2-(2,2,3,3,4,4,4-heptafluorobutoxy) ethoxy)ethoxy)ethyl)isoindoline-1,3-dione.

Figure S24: The ^19^FNMR spectra of 2-(2-(2-(2-(2,2,3,3,4,4,4-heptafluorobutoxy) ethoxy)ethoxy)ethyl)isoindoline-1,3-dione.

2-(2-(2-(2,2,3,3,4,4,4-heptafluorobutoxy)ethoxy)ethoxy)ethanamine (**11**)

2-(2-(2-(2-(2,2,3,3,4,4,4-heptafluorobutoxy)ethoxy)ethoxy)ethyl)isoindoline-1,3-dione (1.99 g, 4.32 mmol), hydrazine hydrate (0.73 mL, 15 mmol) and ethanol (120 mL) were stirred at 95 ^o^C for overnight. After cooling to room temperature, the ethanol was evaporated by rotary evaporation, the residue was diluted with CH_2_Cl_2_ and washed with 10% NaOH. Aqueous layers were combined and extracted with CH_2_Cl_2_. The combined organic layers were washed with brine and dried over Na_2_SO_4_. The removal of CH_2_Cl_2_ afforded yellow oil (1.3 g, 91%) as amine product which was used without further purification.

4,9-dibromo-2,7-bis(2-(2-(2-(2,2,3,3,4,4,4-heptafluorobutoxy)ethoxy)ethoxy)ethyl)benzo[lmn][3,8]phenanthroline-1,3,6,8(2H,7H)-tetraone (**NDI-3O-7F**)

2-(2-(2-(2,2,3,3,4,4,4-heptafluorobutoxy)ethoxy)ethoxy)ethanamine (1300 mg, 3.92 mmol, 4 eq) was added to a suspension of 2,6-dibromo-1,4,5,8-naphthalene tetracarboxylic diimide (384 mg, 0.9 mmol, 1 eq) in 15 mL of glacial acetic acid and heated to 140 ^o^C for 1 h. The reaction mixture was then cooled to room temperature and added to water, then extracted with CH_2_Cl_2_. The organic phase was washed with brine, dried over anhydrous Na_2_SO_4_ and removed solvent by rotary evaporator. The crude product was purified by silica gel column chromatography with CH_2_Cl_2_/acetone as the eluent afforded target compound as a yellow solid (350 mg, 37%).

^1^HNMR (400 MHz, CDCl_3_): δ (ppm) 8.98 (s, 2H), 4.47 (t, J=5.8, 4H), 3.99 (t, J=14.0, 4H), 3.85 (t, J=5.8, 4H), 3.74-3.67 (m, 8H), 3.66-3.58 (m, 8H).

^13^CNMR (100 MHz, CDCl_3_): δ (ppm) 160.86, 160.77, 139.06, 128.38, 127.80, 125.34, 124.12, 72.30, 70.77, 70.65, 70.10, 68.00, 67.63, 40.01.

^19^FNMR (400 MHz, CDCl_3_): δ (ppm) -80.95 (t, J= 9.7 Hz), -120.76 — -120.80 (m), -127.80.

HRMS Calcd. for C_34_H_30_Br_2_F_14_N_2_O_10_Na [M+Na]^+^ m/z: 1074.99209, found: 1074.99030.

Figure S25: The ^1^HNMR spectra of 4,9-dibromo-2,7-bis(2-(2-(2-(2,2,3,3,4,4,4- heptafluorobutoxy)ethoxy)ethoxy)ethyl)benzo[lmn][3,8]phenanthroline-1,3,6,8(2H,7H)-tetraone.

Figure S26: The ^19^FNMR spectra of 4,9-dibromo-2,7-bis(2-(2-(2-(2,2,3,3,4,4,4- heptafluorobutoxy)ethoxy)ethoxy)ethyl)benzo[lmn][3,8]phenanthroline-1,3,6,8(2H,7H)-tetraone.

Figure S27: The ^13^CNMR spectra of 4,9-dibromo-2,7-bis(2-(2-(2-(2,2,3,3,4,4,4- heptafluorobutoxy)ethoxy)ethoxy)ethyl)benzo[lmn][3,8]phenanthroline-1,3,6,8(2H,7H)-tetraone.

Figure S28: The HRMS spectra of 4,9-dibromo-2,7-bis(2-(2-(2-(2,2,3,3,4,4,4- heptafluorobutoxy)ethoxy)ethoxy)ethyl)benzo[lmn][3,8]phenanthroline-1,3,6,8(2H,7H)-tetraone.

**General synthetic procedures for the NDI based polymers**

Scheme S4. Synthetic route to NDI based conjugated polymers P-3O-xF.

To a dry three-neck flask, NDI-based monomer (0.1 mmol) and dialkoxybithiazole based monomer (0.1mmol) were added under argon followed by tris(dibenzylideneacetone) dipalladium [Pd_2_(dba)_3_](8 mg) and tri(o-tolyl)phosphine [P(o-tolyl)_3_](12 mg). The flask and its contents were subjected to 3 pump/purge cycles with N_2_ followed by addition of anhydrous, degassed chlorobenzene (5 mL) via syringe. The reaction mixture was stirred at 110 ℃ for 24 hours. After cooling to room temperature, the deeply colored reaction mixture was dropped into 100 mL vigorously stirred methanol (containing 5 mL 12 M hydrochloride acid). After stirring for 4 hours, the precipitated solid was collected by filtration. The solid polymers were re-dissolved in chloroform and reprecipitated into methanol. After filtration, the polymers were subjected to sequential Soxhlet extraction. The sequential solvents were methanol, hexane and chloroform. Impurities and low-molecular-weight fraction were removed by methanol. Finally, the polymer solution in chloroform was concentrated to give the polymer as a dark solid.

**P-3O-3F** Synthesis according to the general polymerization procedure: monomer NDI-3O-3F (85.2 mg, 0.1 mmol), monomer 2Tz (75 mg, 0.1 mmol), dry chlorobenzene (5 mL). The polymer was obtained as a dark solid (110 mg, 94 %).

^1^HNMR (500 MHz, CDCl_3_): δ (ppm) 10.38-8.84 (m, 2H), 5.27-3.10 (m, 32H), 2.27-0.39 (m, 30H). GPC: Mn = 23.3 kDa, Mw = 55.1 kDa, PDI = 2.36.

**P-3O-5F** Synthesis according to the general polymerization procedure: monomer NDI-3O-5F (86.5 mg, 0.1 mmol), monomer 2Tz (75 mg, 0.1 mmol), dry chlorobenzene (5 mL). The polymer was obtained as a dark solid (107 mg, 95 %).

^1^HNMR (500 MHz, CDCl_3_): δ (ppm) 10.39-9.03 (m, 2H), 5.31-2.95 (m, 32H), 2.08-0.51 (m, 30H). GPC: Mn = 29.7 kDa, Mw = 67.5 kDa, PDI = 2.27.

**P-3O-7F** Synthesis according to the general polymerization procedure: monomer NDI-3O-7F (110 mg, 0.1 mmol), monomer 2Tz (75 mg, 0.1 mmol), dry chlorobenzene (5 mL). The polymer was obtained as a dark solid (60 mg, 46 %).

^1^HNMR (500 MHz, CDCl_3_): δ (ppm) 9.35-8.45 (br, 2H), 5.04-2.79 (m, 32H), 2.08-0.71 (m, 30H). GPC: Mn = 31.1 kDa, Mw = 88.0 kDa, PDI = 2.82.

Figure S29. GPC trace of P-3O-3F, P-3O-5F, and P-3O-7F.

Figure S30. ^1^HNMR spectra of P-3O-3F, P-3O-5F, and P-3O-7F.

**Synthetic route for the NDI based P-3O-7H polymers**

Scheme S5. General synthetic route to NDI based conjugated polymers P-3O-7H.

2-(2-(2-butoxyethoxy)ethoxy)ethyl 4-methylbenzenesulfonate

To a stirred solution of 2-(2-(2-butoxyethoxy)ethoxy)ethanol (11.34 g, 55 mmol) in THF (50 mL) at 0 ^o^C, a solution of NaOH (3000 mg, 75 mmol) dissolved in water (30 mL) was added, the resulting mixture was stirring for 2 hours at 0 ^o^C. Then, a solution of *p*-toluenesulfonyl chloride (9.53 g, 50 mmol) in THF (50 mL) was added drop-wise. The reaction mixture was allowed to warm to room temperature and stirred overnight. After workup, the reaction mixture was washed with brine for three times, dried over anhydrous Na_2_SO_4_, filtered, concentrated, and dried under vacuum to afford the crude product as a white solid (16.96 g, 94%). The crude product was used in the next reaction without further purification.

^1^HNMR (400 MHz, CDCl_3_): δ (ppm) 7.81 (d, J=8.2, 2H), 7.35 (d, J=8.1, 2H), 4.16 (t, 2H), 3.79-3.51 (m, 10H), 3.48-3.37 (m, 2H), 2.44 (s, 3H), 1.63-1.49 (m, 2H), 1.40-1.31 (m, 2H), 0.91 (t, 3H).

Figure S31: The ^1^HNMR spectra of 2-(2-(2-butoxyethoxy)ethoxy)ethyl 4-methylbenzenesulfonate.

2-(2-(2-(2-(pentyloxy)ethoxy)ethoxy)ethyl)isoindoline-1,3-dione

2-(2-(2-butoxyethoxy)ethoxy)ethyl 4-methylbenzenesulfonate (7.2 g, 20 mmol) and potassium phthalimide (4.46 g, 24 mmol) were dissolved in DMF (15 mL). The reaction mixture was stirred at 100 ^o^C overnight. After cooling to room temperature, deionized water was added and the mixture was extracted by ethyl acetate, the organic phase was collected, dried over anhydrous Na_2_SO_4_, filtered, concentrated. The crude product was purified by silica gel column chromatography with dichlorormethane/ethyl acetate (1:1) as the eluent to afford target compound as a colorless oil (3.7 g, 55%).

^1^HNMR (400 MHz, CDCl_3_): δ (ppm) 7.84 (dd, J=5.4, 3.1, 2H), 7.71 (dd, J=5.5, 3.1, 2H), 3.89 (t, 2H), 3.73 (t, 2H), 3.67-3.46 (m, 8H), 3.41 (t, 2H), 1.56-1.47 (m, 2H), 1.39-1.27 (m, 2H), 0.89 (t, 3H).

^13^CNMR (100 MHz, CDCl_3_): δ (ppm) 168.24, 133.89, 132.17, 123.21, 71.16, 70.65, 70.57, 70.14, 70.02, 67.90, 37.28, 31.69, 19.25, 13.91.

Figure S32: The ^1^HNMR spectra of 2-(2-(2-(2-(pentyloxy)ethoxy)ethoxy)ethyl)isoindoline-1,3-dione.

Figure S33: The ^13^CNMR spectra of 2-(2-(2-(2-(pentyloxy)ethoxy)ethoxy)ethyl)isoindoline-1,3-dione.

2-(2-(2-butoxyethoxy)ethoxy)ethanamine

2-(2-(2-(2-(pentyloxy)ethoxy)ethoxy)ethyl)isoindoline-1,3-dione (3.7 g, 11 mmol), hydrazine hydrate (1.88 mL, 38.6 mmol) and 150 mL ethanol were stirred at 95 ^o^C for overnight. Then, 2 mL con. HCl was added and refluxed for another hour. After cooling to room temperature, the ethanol was evaporated by rotary evaporation, the residue was diluted with CH_2_Cl_2_ and washed with 10% NaOH. Aqueous layers were combined and extracted with CH_2_Cl_2_. The combined organic layers were washed with brine and dried over Na_2_SO_4_. The removal of CH_2_Cl_2_ afforded yellow oil as amine product (2.34 g, 98%) which was used without further purification.

4,9-dibromo-2,7-bis(2-(2-(2-butoxyethoxy)ethoxy)ethyl)benzo[lmn][3,8]phenanthroline-1,3,6,8(2H,7H)-tetraone

2-(2-(2-butoxyethoxy)ethoxy)ethanamine (820 mg, 4 mmol, 4 eq) was added to a suspension of 2,6-dibromo-1,4,5,8-naphthalene tetracarboxylic diimide (426 mg, 1 mmol, 1 eq) in 10 mL of glacial acetic acid and heated to 140 ^o^C for 0.5 h. The reaction mixture was then cooled to room temperature and added to water, then extracted with CH_2_Cl_2_. The organic phase was washed with brine, dried over anhydrous Na_2_SO_4_ and removed solvent by rotary evaporator. The crude product was purified by silica gel column chromatography with CH_2_Cl_2_/acetone as the eluent afforded target compound as a light red solid (254 mg, 32%).

^1^HNMR (400 MHz, CDCl_3_): δ (ppm) 8.98 (s, 2H), 4.47 (t, 4H), 3.86 (t, 4H), 3.73-3.66 (m, 4H), 3.66-3.61 (m, 4H), 3.59-3.57 (m, 4H), 3.52-3.49 (m, 4H), 1.56-1.49 (m, 4H), 1.37-1.29 (m, 4H), 0.89 (t, 6H).

^13^CNMR (125 MHz, CDCl_3_): δ (ppm) 160.87, 160.77, 139.07, 128.38, 127.80, 125.36, 124.14, 71.21, 70.64, 70.13, 70.03, 67.59, 40.04, 31.68, 19.26, 13.93.

Figure S34: The ^1^HNMR spectra of 4,9-dibromo-2,7-bis(2-(2-(2-butoxyethoxy)ethoxy)ethyl)benzo[lmn][3,8]phenanthroline-1,3,6,8(2H,7H)-tetraone.

Figure S35: The ^13^CNMR spectra of 4,9-dibromo-2,7-bis(2-(2-(2-butoxyethoxy)ethoxy)ethyl)benzo[lmn][3,8]phenanthroline-1,3,6,8(2H,7H)-tetraone.

**Synthetic procedures for the NDI based P-3O-7H polymers**

Scheme S6. Polymerization synthetic route to NDI based conjugated polymers P-3O-7H

To a dry three-neck flask, NDI-3O-7H monomer (80 mg, 0.1 mmol) and dialkoxybithiazole based monomer (75 mg, 0.1mmol) were added under argon followed by tris(dibenzylideneacetone) dipalladium [Pd_2_(dba)_3_] (8 mg), tri(o-tolyl)phosphine [P(o-tolyl)_3_](12 mg) and CuI (4 mg). The flask and its contents were subjected to 3 pump/purge cycles with N_2_ followed by addition of anhydrous, degassed chlorobenzene (5 mL) via syringe. The reaction mixture was stirred at 110 ℃ for 48 hours. After cooling to room temperature, the deeply colored reaction mixture was dropped into 100 mL vigorously stirred methanol (containing 5 mL 12 M hydrochloride acid). After stirring for 1 hour, the precipitated solid was collected by filtration. The solid polymers were re-dissolved in chloroform and reprecipitated into methanol. After filtration, the polymers were subjected to sequential Soxhlet extraction. The sequential solvents were methanol, hexane, acetone and chloroform. Impurities and low-molecular-weight fraction were removed by methanol. Finally, the polymer solution in chloroform was concentrated to give the polymer P-3O-7H (90 mg, 82%) as a dark solid.

**UV-VIS-NIR Absorption Spectroscopy**


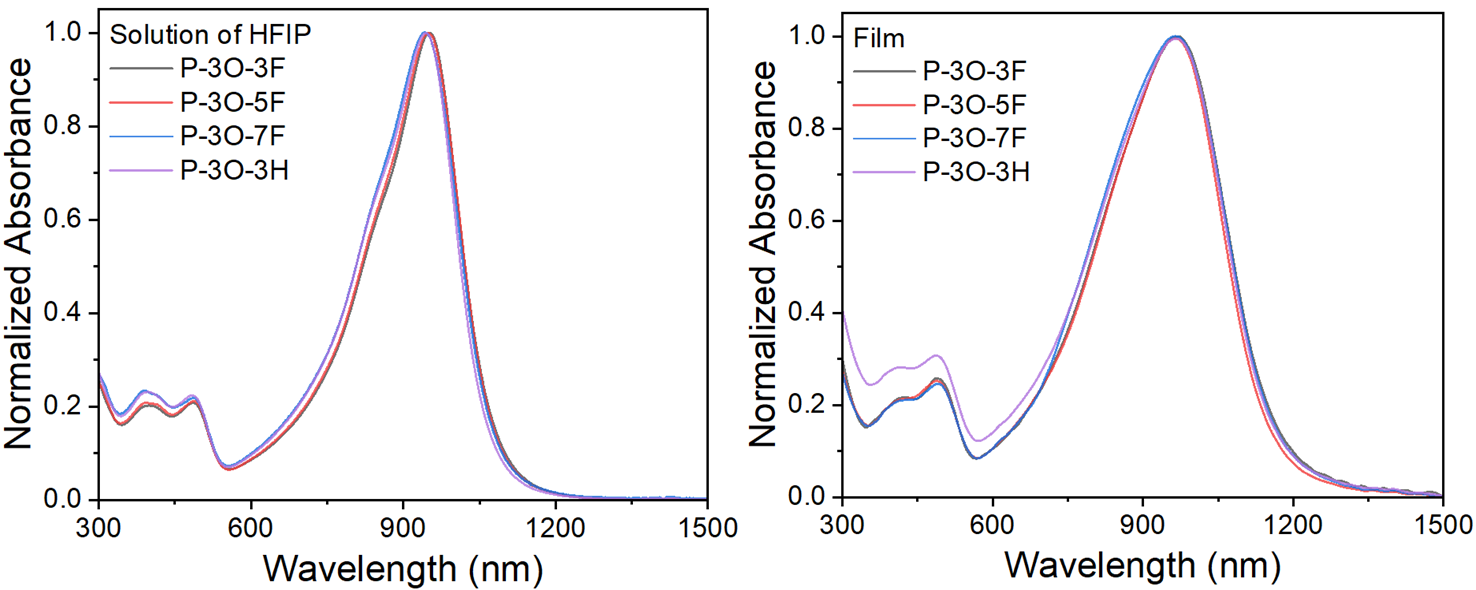


Figure S36. Normalized UV-vis-NIR absorption spectra of P-3O-3F, P-3O-5F, and P-3O-7F and the reference of P-3O-3H in chloroform (10^-5^ M) solution and in a thin film.

**Cyclic Voltammetry**

Figure S37. Cyclic Voltammetry of P-3O-3F, P-3O-5F, and P-3O-7F.

**UV-VIS-NIR Absorption Spectroscopy**

**
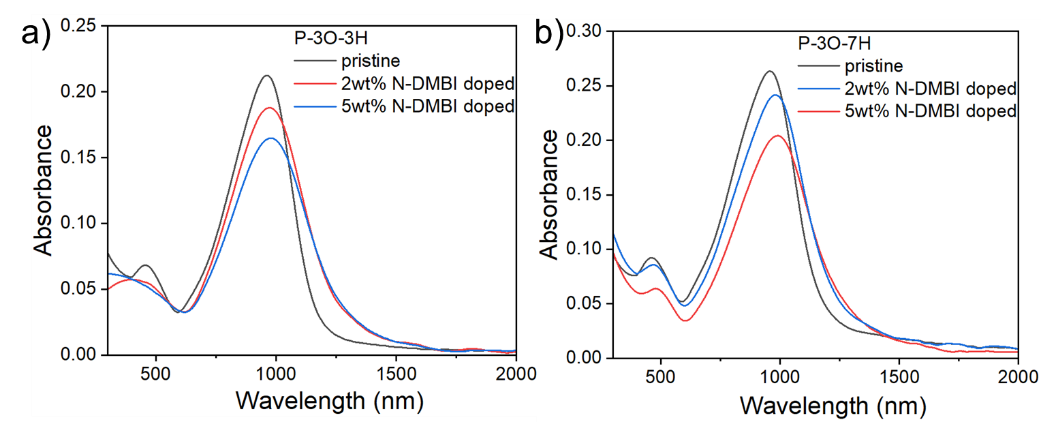
**

Figure S38. UV-vis-NIR thin film absorption spectra of the pristine and N-DMBI doped a) P-3O-3H and b) P-3O-7H.

**AFM height images**


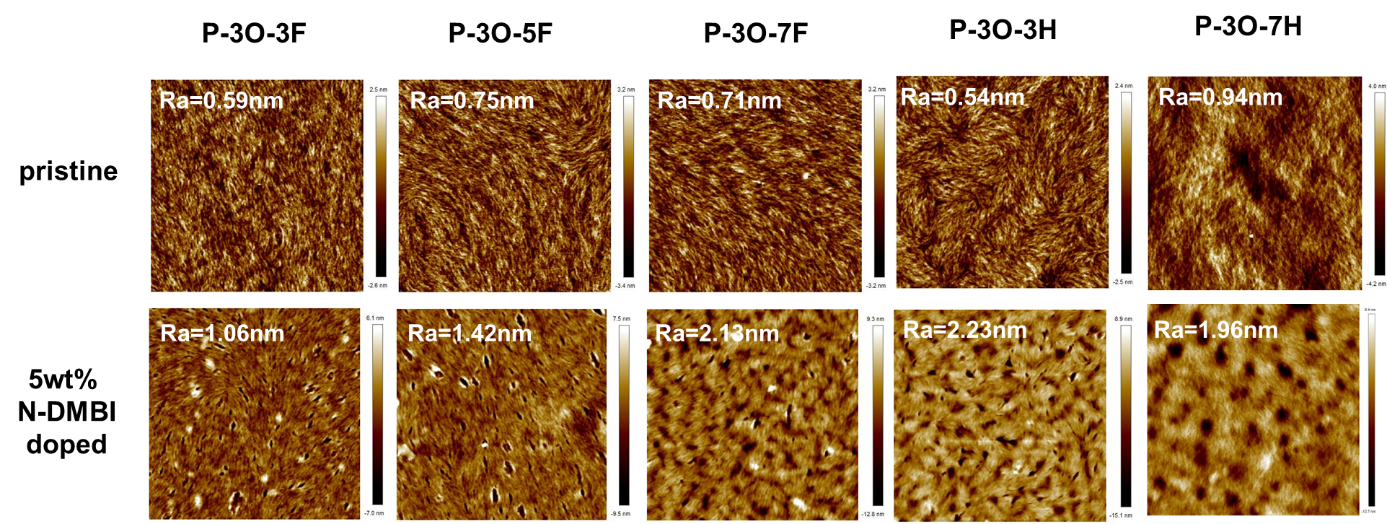


Figure S39. AFM height images of pristine and 5 wt% doped P-3O-3F, P-3O-5F, and P-3O-7F and the reference of P-3O-3H, P-3O-7H films.

**2D GIWAXS patterns and linecuts**


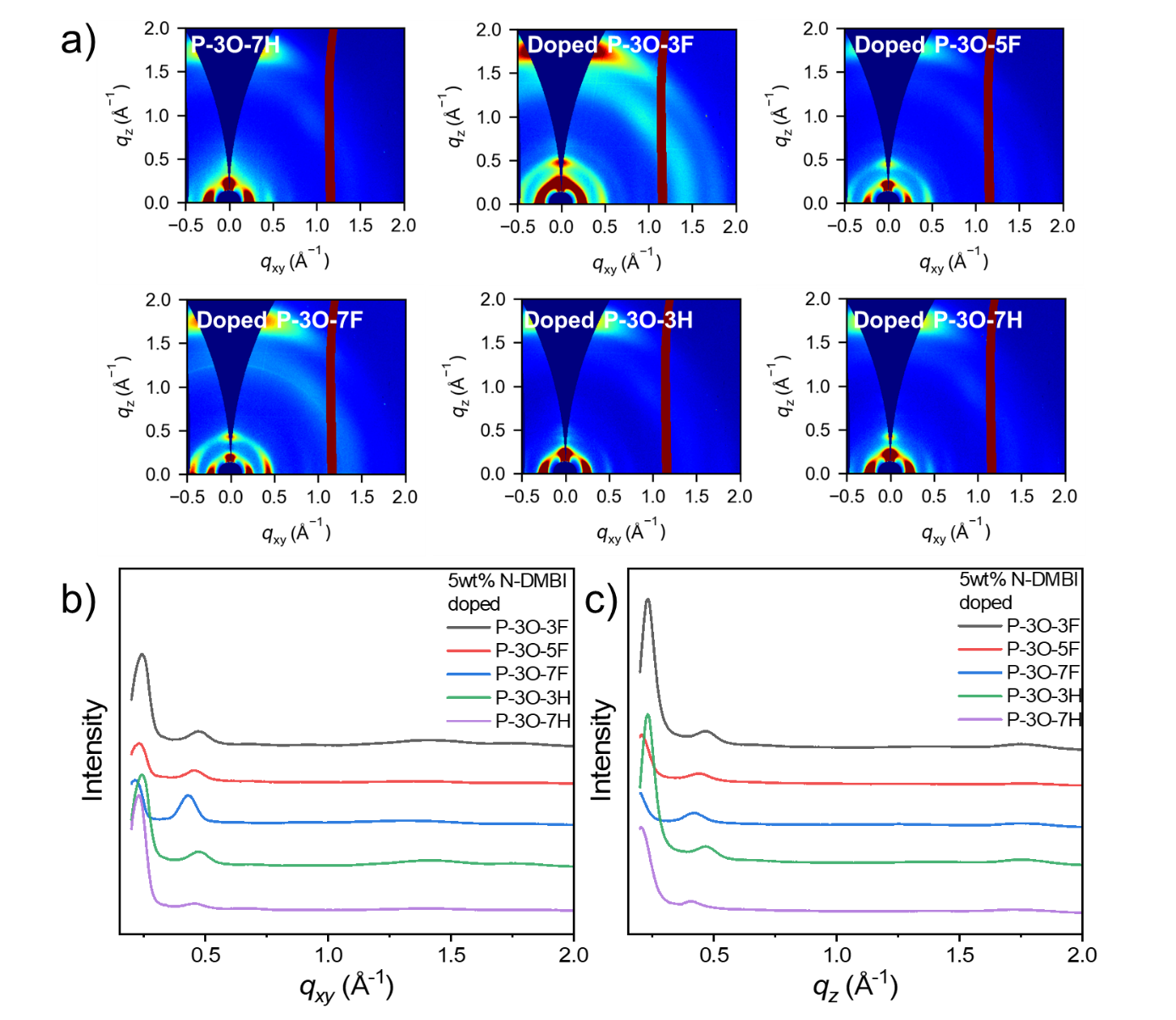


Figure S40. a) 2D GIWAXS patterns of the intrinsic P-3O-7H and 5wt% N-DMBI doped of P-3O-xH and P-3O-xF (x=3-7). Linecuts of b) *q_xy_* and c) *q_z_* of 5wt% N-DMBI doped of P-3O-xH and P-3O-xF (x=3-7).

Table S1. Summary of GIWAXS results parameter of NDI based polymers.

|  | d-spacing  (Å) | π-π spacing  (Å) | Lc ^OOP^ (100)  (nm) | Lc^IP^ (010)  (nm) | g_lam_ | g_π-π_ |
| --- | --- | --- | --- | --- | --- | --- |
| P-3O-3F | 26.18 | 4.41 | 8.87 | 1.81 | 0.217 | 0.197 |
| doped  P-3O-3F | 25.86 | 4.44 | 8.11 | 1.74 | 0.225 | 0.202 |
| P-3O-5F | 28.05 | 4.61 | 9.20 | 1.58 | 0.220 | 0.216 |
| doped  P-3O-5F | 27.32 | 4.62 | 9.76 | 1.57 | 0.211 | 0.216 |
| P-3O-7F | 29.50 | 4.76 | 9.23 | 1.59 | 0.226 | 0.218 |
| doped  P-3O-7F | 29.22 | 4.76 | 9.53 | 1.49 | 0.221 | 0.226 |
| P-3O-3H | 25.65 | 4.42 | 8.40 | 1.53 | 0.220 | 0.214 |
| doped  P-3O-3H | 24.93 | 4.47 | 9.65 | 1.70 | 0.203 | 0.205 |
| P-3O-7H | 27.56 | 4.51 | 8.44 | 1.44 | 0.228 | 0.223 |
| doped  P-3O-7H | 27.44 | 4.50 | 8.91 | 1.65 | 0.221 | 0.208 |

**Water contact angle**


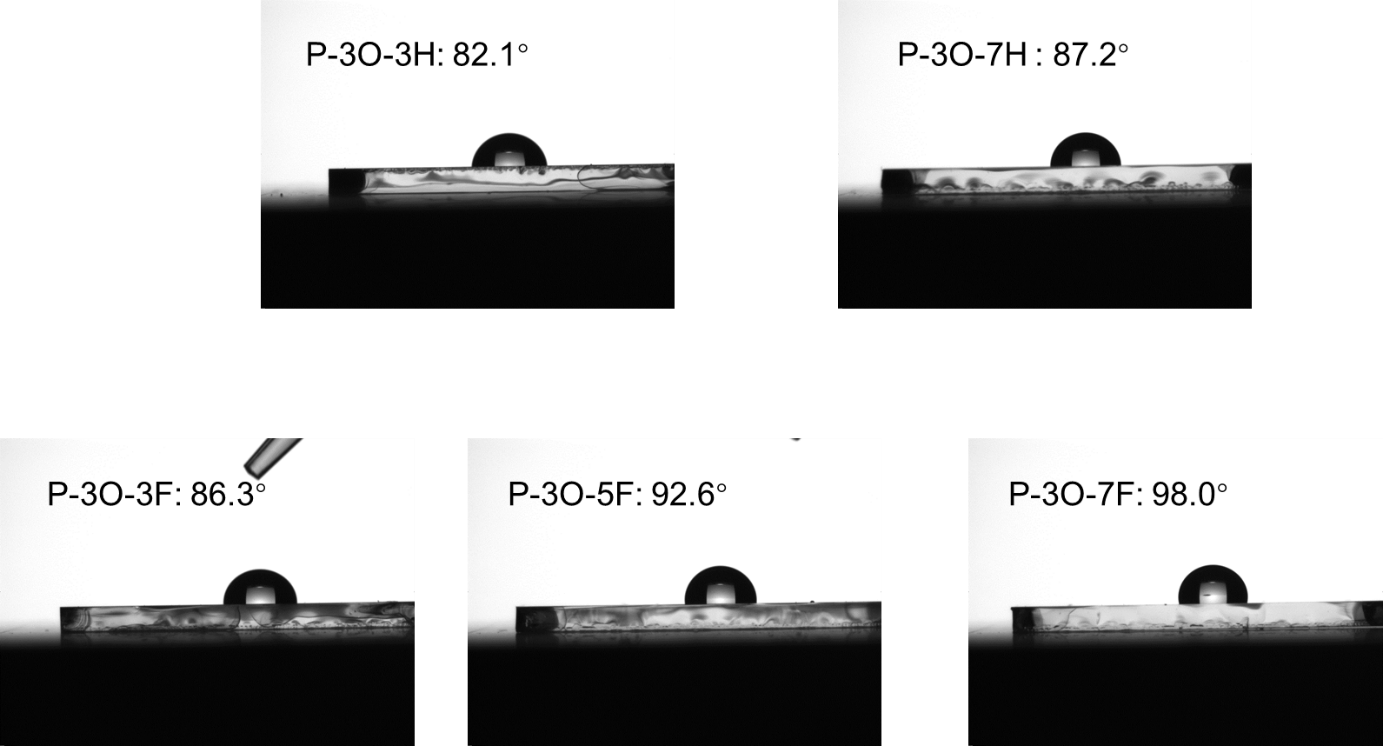


Figure S41. Water contact angle of P-3O-3F, P-3O-5F, and P-3O-7F and the reference of P-3O-3H and P-3O-7H.

**Thermoelectric devices fabrication and measurement**


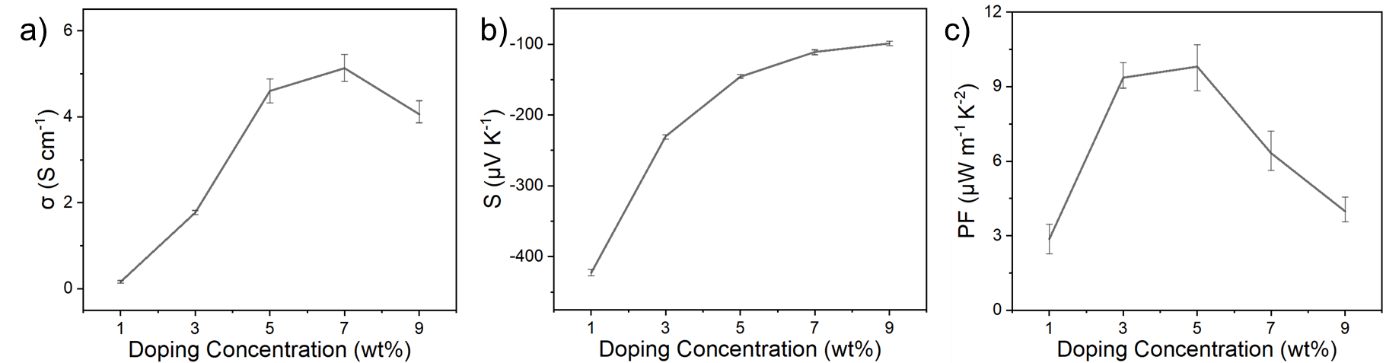


Figure S42 a) Electrical conductivities, b) Seebeck coefficients and c) power factors recorded for P-3O-7H at different doping ratios.
